# Extraction and validation of a new set of CMS pythia8 tunes from underlying-event measurements

**DOI:** 10.1140/epjc/s10052-019-7499-4

**Published:** 2020-01-03

**Authors:** A. M. Sirunyan, A. Tumasyan, W. Adam, F. Ambrogi, E. Asilar, T. Bergauer, J. Brandstetter, M. Dragicevic, J. Erö, A. Escalante Del Valle, M. Flechl, R. Frühwirth, V. M. Ghete, J. Hrubec, M. Jeitler, N. Krammer, I. Krätschmer, D. Liko, T. Madlener, I. Mikulec, N. Rad, H. Rohringer, J. Schieck, R. Schöfbeck, M. Spanring, D. Spitzbart, W. Waltenberger, J. Wittmann, C.-E. Wulz, M. Zarucki, V. Chekhovsky, V. Mossolov, J. Suarez Gonzalez, E. A. De Wolf, D. Di Croce, X. Janssen, J. Lauwers, M. Pieters, H. Van Haevermaet, P. Van Mechelen, N. Van Remortel, S. Abu Zeid, F. Blekman, J. D’Hondt, J. De Clercq, K. Deroover, G. Flouris, D. Lontkovskyi, S. Lowette, I. Marchesini, S. Moortgat, L. Moreels, Q. Python, K. Skovpen, S. Tavernier, W. Van Doninck, P. Van Mulders, I. Van Parijs, D. Beghin, B. Bilin, H. Brun, B. Clerbaux, G. De Lentdecker, H. Delannoy, B. Dorney, G. Fasanella, L. Favart, R. Goldouzian, A. Grebenyuk, A. K. Kalsi, T. Lenzi, J. Luetic, N. Postiau, E. Starling, L. Thomas, C. Vander Velde, P. Vanlaer, D. Vannerom, Q. Wang, T. Cornelis, D. Dobur, A. Fagot, M. Gul, I. Khvastunov, D. Poyraz, C. Roskas, D. Trocino, M. Tytgat, W. Verbeke, B. Vermassen, M. Vit, N. Zaganidis, H. Bakhshiansohi, O. Bondu, S. Brochet, G. Bruno, C. Caputo, P. David, C. Delaere, M. Delcourt, A. Giammanco, G. Krintiras, V. Lemaitre, A. Magitteri, K. Piotrzkowski, A. Saggio, M. Vidal Marono, P. Vischia, S. Wertz, J. Zobec, F. L. Alves, G. A. Alves, M. Correa Martins Junior, G. Correia Silva, C. Hensel, A. Moraes, M. E. Pol, P. Rebello Teles, E. Belchior Batista Das Chagas, W. Carvalho, J. Chinellato, E. Coelho, E. M. Da Costa, G. G. Da Silveira, D. De Jesus Damiao, C. De Oliveira Martins, S. Fonseca De Souza, H. Malbouisson, D. Matos Figueiredo, M. Melo De Almeida, C. Mora Herrera, L. Mundim, H. Nogima, W. L. Prado Da Silva, L. J. Sanchez Rosas, A. Santoro, A. Sznajder, M. Thiel, E. J. Tonelli Manganote, F. Torres Da Silva De Araujo, A. Vilela Pereira, S. Ahuja, C. A. Bernardes, L. Calligaris, T. R. Fernandez Perez Tomei, E. M. Gregores, P. G. Mercadante, S. F. Novaes, SandraS. Padula, A. Aleksandrov, R. Hadjiiska, P. Iaydjiev, A. Marinov, M. Misheva, M. Rodozov, M. Shopova, G. Sultanov, A. Dimitrov, L. Litov, B. Pavlov, P. Petkov, W. Fang, X. Gao, L. Yuan, M. Ahmad, J. G. Bian, G. M. Chen, H. S. Chen, M. Chen, Y. Chen, C. H. Jiang, D. Leggat, H. Liao, Z. Liu, S. M. Shaheen, A. Spiezia, J. Tao, Z. Wang, E. Yazgan, H. Zhang, S. Zhang, J. Zhao, Y. Ban, G. Chen, A. Levin, J. Li, L. Li, Q. Li, Y. Mao, S. J. Qian, D. Wang, Y. Wang, C. Avila, A. Cabrera, C. A. Carrillo Montoya, L. F. Chaparro Sierra, C. Florez, C. F. González Hernández, M. A. Segura Delgado, B. Courbon, N. Godinovic, D. Lelas, I. Puljak, T. Sculac, Z. Antunovic, M. Kovac, V. Brigljevic, D. Ferencek, K. Kadija, B. Mesic, M. Roguljic, A. Starodumov, T. Susa, M. W. Ather, A. Attikis, M. Kolosova, G. Mavromanolakis, J. Mousa, C. Nicolaou, F. Ptochos, P. A. Razis, H. Rykaczewski, M. Finger, M. Finger, E. Ayala, E. Carrera Jarrin, A. Mahrous, Y. Mohammed, E. Salama, S. Bhowmik, A. Carvalho Antunes De Oliveira, R. K. Dewanjee, K. Ehataht, M. Kadastik, M. Raidal, C. Veelken, P. Eerola, H. Kirschenmann, J. Pekkanen, M. Voutilainen, J. Havukainen, J. K. Heikkilä, T. Järvinen, V. Karimäki, R. Kinnunen, T. Lampén, K. Lassila-Perini, S. Laurila, S. Lehti, T. Lindén, P. Luukka, T. Mäenpää, H. Siikonen, E. Tuominen, J. Tuominiemi, T. Tuuva, M. Besancon, F. Couderc, M. Dejardin, D. Denegri, J. L. Faure, F. Ferri, S. Ganjour, A. Givernaud, P. Gras, G. Hamel de Monchenault, P. Jarry, C. Leloup, E. Locci, J. Malcles, G. Negro, J. Rander, A. Rosowsky, M. Ö. Sahin, M. Titov, A. Abdulsalam, C. Amendola, I. Antropov, F. Beaudette, P. Busson, C. Charlot, R. Granier de Cassagnac, I. Kucher, A. Lobanov, J. Martin Blanco, C. Martin Perez, M. Nguyen, C. Ochando, G. Ortona, P. Paganini, J. Rembser, R. Salerno, J. B. Sauvan, Y. Sirois, A. G. Stahl Leiton, A. Zabi, A. Zghiche, J.-L. Agram, J. Andrea, D. Bloch, J.-M. Brom, E. C. Chabert, V. Cherepanov, C. Collard, E. Conte, J.-C. Fontaine, D. Gelé, U. Goerlach, M. Jansová, A.-C. Le Bihan, N. Tonon, P. Van Hove, S. Gadrat, S. Beauceron, C. Bernet, G. Boudoul, N. Chanon, R. Chierici, D. Contardo, P. Depasse, H. El Mamouni, J. Fay, L. Finco, S. Gascon, M. Gouzevitch, G. Grenier, B. Ille, F. Lagarde, I. B. Laktineh, H. Lattaud, M. Lethuillier, L. Mirabito, S. Perries, A. Popov, V. Sordini, G. Touquet, M. Vander Donckt, S. Viret, T. Toriashvili, Z. Tsamalaidze, C. Autermann, L. Feld, M. K. Kiesel, K. Klein, M. Lipinski, M. Preuten, M. P. Rauch, C. Schomakers, J. Schulz, M. Teroerde, B. Wittmer, A. Albert, D. Duchardt, M. Erdmann, S. Erdweg, T. Esch, R. Fischer, S. Ghosh, A. Güth, T. Hebbeker, C. Heidemann, K. Hoepfner, H. Keller, L. Mastrolorenzo, M. Merschmeyer, A. Meyer, P. Millet, S. Mukherjee, T. Pook, M. Radziej, H. Reithler, M. Rieger, A. Schmidt, D. Teyssier, S. Thüer, G. Flügge, O. Hlushchenko, T. Kress, T. Müller, A. Nehrkorn, A. Nowack, C. Pistone, O. Pooth, D. Roy, H. Sert, A. Stahl, M. Aldaya Martin, T. Arndt, C. Asawatangtrakuldee, I. Babounikau, K. Beernaert, O. Behnke, U. Behrens, A. Bermúdez Martínez, D. Bertsche, A. A. Bin Anuar, K. Borras, V. Botta, A. Campbell, P. Connor, C. Contreras-Campana, V. Danilov, A. De Wit, M. M. Defranchis, C. Diez Pardos, D. Domínguez Damiani, G. Eckerlin, T. Eichhorn, A. Elwood, E. Eren, E. Gallo, A. Geiser, J. M. Grados Luyando, A. Grohsjean, M. Guthoff, M. Haranko, A. Harb, H. Jung, M. Kasemann, J. Keaveney, C. Kleinwort, J. Knolle, D. Krücker, W. Lange, A. Lelek, T. Lenz, J. Leonard, K. Lipka, W. Lohmann, R. Mankel, I.-A. Melzer-Pellmann, A. B. Meyer, M. Meyer, M. Missiroli, J. Mnich, V. Myronenko, S. K. Pflitsch, D. Pitzl, A. Raspereza, P. Saxena, P. Schütze, C. Schwanenberger, R. Shevchenko, A. Singh, H. Tholen, O. Turkot, A. Vagnerini, M. Van De Klundert, G. P. Van Onsem, R. Walsh, Y. Wen, K. Wichmann, C. Wissing, O. Zenaiev, R. Aggleton, S. Bein, L. Benato, A. Benecke, V. Blobel, T. Dreyer, A. Ebrahimi, E. Garutti, D. Gonzalez, P. Gunnellini, J. Haller, A. Hinzmann, A. Karavdina, G. Kasieczka, R. Klanner, R. Kogler, N. Kovalchuk, S. Kurz, V. Kutzner, J. Lange, D. Marconi, J. Multhaup, M. Niedziela, C. E. N. Niemeyer, D. Nowatschin, A. Perieanu, A. Reimers, O. Rieger, C. Scharf, P. Schleper, S. Schumann, J. Schwandt, J. Sonneveld, H. Stadie, G. Steinbrück, F. M. Stober, M. Stöver, B. Vormwald, I. Zoi, M. Akbiyik, C. Barth, M. Baselga, S. Baur, E. Butz, R. Caspart, T. Chwalek, F. Colombo, W. De Boer, A. Dierlamm, K. El Morabit, N. Faltermann, B. Freund, M. Giffels, M. A. Harrendorf, F. Hartmann, S. M. Heindl, U. Husemann, I. Katkov, S. Kudella, S. Mitra, M. U. Mozer, Th. Müller, M. Musich, M. Plagge, G. Quast, K. Rabbertz, M. Schröder, I. Shvetsov, H. J. Simonis, R. Ulrich, S. Wayand, M. Weber, T. Weiler, C. Wöhrmann, R. Wolf, G. Anagnostou, G. Daskalakis, T. Geralis, A. Kyriakis, D. Loukas, G. Paspalaki, A. Agapitos, G. Karathanasis, P. Kontaxakis, A. Panagiotou, I. Papavergou, N. Saoulidou, E. Tziaferi, K. Vellidis, K. Kousouris, I. Papakrivopoulos, G. Tsipolitis, I. Evangelou, C. Foudas, P. Gianneios, P. Katsoulis, P. Kokkas, S. Mallios, N. Manthos, I. Papadopoulos, E. Paradas, J. Strologas, F. A. Triantis, D. Tsitsonis, M. Bartók, M. Csanad, N. Filipovic, P. Major, M. I. Nagy, G. Pasztor, O. Surányi, G. I. Veres, G. Bencze, C. Hajdu, D. Horvath, Á Hunyadi, F. Sikler, T. Á Vámi, V. Veszpremi, G. Vesztergombi, N. Beni, S. Czellar, J. Karancsi, A. Makovec, J. Molnar, Z. Szillasi, P. Raics, Z. L. Trocsanyi, B. Ujvari, S. Choudhury, J. R. Komaragiri, P. C. Tiwari, S. Bahinipati, C. Kar, P. Mal, K. Mandal, A. Nayak, S. Roy Chowdhury, D. K. Sahoo, S. K. Swain, S. Bansal, S. B. Beri, V. Bhatnagar, S. Chauhan, R. Chawla, N. Dhingra, S. K. Gill, R. Gupta, A. Kaur, M. Kaur, P. Kumari, M. Lohan, M. Meena, A. Mehta, K. Sandeep, S. Sharma, J. B. Singh, A. K. Virdi, G. Walia, A. Bhardwaj, B. C. Choudhary, R. B. Garg, M. Gola, S. Keshri, Ashok Kumar, S. Malhotra, M. Naimuddin, P. Priyanka, K. Ranjan, Aashaq Shah, R. Sharma, R. Bhardwaj, M. Bharti, R. Bhattacharya, S. Bhattacharya, U. Bhawandeep, D. Bhowmik, S. Dey, S. Dutt, S. Dutta, S. Ghosh, K. Mondal, S. Nandan, A. Purohit, P. K. Rout, A. Roy, G. Saha, S. Sarkar, M. Sharan, B. Singh, S. Thakur, P. K. Behera, A. Muhammad, R. Chudasama, D. Dutta, V. Jha, V. Kumar, D. K. Mishra, P. K. Netrakanti, L. M. Pant, P. Shukla, P. Suggisetti, T. Aziz, M. A. Bhat, S. Dugad, G. B. Mohanty, N. Sur, RavindraKumar Verma, S. Banerjee, S. Bhattacharya, S. Chatterjee, P. Das, M. Guchait, Sa. Jain, S. Karmakar, S. Kumar, M. Maity, G. Majumder, K. Mazumdar, N. Sahoo, T. Sarkar, S. Chauhan, S. Dube, V. Hegde, A. Kapoor, K. Kothekar, S. Pandey, A. Rane, A. Rastogi, S. Sharma, S. Chenarani, E. Eskandari Tadavani, S. M. Etesami, M. Khakzad, M. Mohammadi Najafabadi, M. Naseri, F. Rezaei Hosseinabadi, B. Safarzadeh, M. Zeinali, M. Felcini, M. Grunewald, M. Abbrescia, C. Calabria, A. Colaleo, D. Creanza, L. Cristella, N. De Filippis, M. De Palma, A. Di Florio, F. Errico, L. Fiore, A. Gelmi, G. Iaselli, M. Ince, S. Lezki, G. Maggi, M. Maggi, G. Miniello, S. My, S. Nuzzo, A. Pompili, G. Pugliese, R. Radogna, A. Ranieri, G. Selvaggi, A. Sharma, L. Silvestris, R. Venditti, P. Verwilligen, G. Abbiendi, C. Battilana, D. Bonacorsi, L. Borgonovi, S. Braibant-Giacomelli, R. Campanini, P. Capiluppi, A. Castro, F. R. Cavallo, S. S. Chhibra, G. Codispoti, M. Cuffiani, G. M. Dallavalle, F. Fabbri, A. Fanfani, E. Fontanesi, P. Giacomelli, C. Grandi, L. Guiducci, F. Iemmi, S. Lo Meo, S. Marcellini, G. Masetti, A. Montanari, F. L. Navarria, A. Perrotta, F. Primavera, A. M. Rossi, T. Rovelli, G. P. Siroli, N. Tosi, S. Albergo, A. Di Mattia, R. Potenza, A. Tricomi, C. Tuve, G. Barbagli, K. Chatterjee, V. Ciulli, C. Civinini, R. D’Alessandro, E. Focardi, G. Latino, P. Lenzi, M. Meschini, S. Paoletti, L. Russo, G. Sguazzoni, D. Strom, L. Viliani, L. Benussi, S. Bianco, F. Fabbri, D. Piccolo, F. Ferro, R. Mulargia, E. Robutti, S. Tosi, A. Benaglia, A. Beschi, F. Brivio, V. Ciriolo, S. Di Guida, M. E. Dinardo, S. Fiorendi, S. Gennai, A. Ghezzi, P. Govoni, M. Malberti, S. Malvezzi, D. Menasce, F. Monti, L. Moroni, M. Paganoni, D. Pedrini, S. Ragazzi, T. Tabarelli de Fatis, D. Zuolo, S. Buontempo, N. Cavallo, A. De Iorio, A. Di Crescenzo, F. Fabozzi, F. Fienga, G. Galati, A. O. M. Iorio, L. Lista, S. Meola, P. Paolucci, C. Sciacca, E. Voevodina, P. Azzi, N. Bacchetta, D. Bisello, A. Boletti, A. Bragagnolo, R. Carlin, P. Checchia, M. Dall’Osso, P. De Castro Manzano, T. Dorigo, U. Dosselli, F. Gasparini, U. Gasparini, A. Gozzelino, S. Y. Hoh, S. Lacaprara, P. Lujan, M. Margoni, A. T. Meneguzzo, J. Pazzini, M. Presilla, P. Ronchese, R. Rossin, F. Simonetto, A. Tiko, E. Torassa, M. Tosi, M. Zanetti, P. Zotto, G. Zumerle, A. Braghieri, A. Magnani, P. Montagna, S. P. Ratti, V. Re, M. Ressegotti, C. Riccardi, P. Salvini, I. Vai, P. Vitulo, M. Biasini, G. M. Bilei, C. Cecchi, D. Ciangottini, L. Fanò, P. Lariccia, R. Leonardi, E. Manoni, G. Mantovani, V. Mariani, M. Menichelli, A. Rossi, A. Santocchia, D. Spiga, K. Androsov, P. Azzurri, G. Bagliesi, L. Bianchini, T. Boccali, L. Borrello, R. Castaldi, M. A. Ciocci, R. Dell’Orso, G. Fedi, F. Fiori, L. Giannini, A. Giassi, M. T. Grippo, F. Ligabue, E. Manca, G. Mandorli, A. Messineo, F. Palla, A. Rizzi, G. Rolandi, P. Spagnolo, R. Tenchini, G. Tonelli, A. Venturi, P. G. Verdini, L. Barone, F. Cavallari, M. Cipriani, D. Del Re, E. Di Marco, M. Diemoz, S. Gelli, E. Longo, B. Marzocchi, P. Meridiani, G. Organtini, F. Pandolfi, R. Paramatti, F. Preiato, S. Rahatlou, C. Rovelli, F. Santanastasio, N. Amapane, R. Arcidiacono, S. Argiro, M. Arneodo, N. Bartosik, R. Bellan, C. Biino, A. Cappati, N. Cartiglia, F. Cenna, S. Cometti, M. Costa, R. Covarelli, N. Demaria, B. Kiani, C. Mariotti, S. Maselli, E. Migliore, V. Monaco, E. Monteil, M. Monteno, M. M. Obertino, L. Pacher, N. Pastrone, M. Pelliccioni, G. L. Pinna Angioni, A. Romero, M. Ruspa, R. Sacchi, R. Salvatico, K. Shchelina, V. Sola, A. Solano, D. Soldi, A. Staiano, S. Belforte, V. Candelise, M. Casarsa, F. Cossutti, A. Da Rold, G. Della Ricca, F. Vazzoler, A. Zanetti, D. H. Kim, G. N. Kim, M. S. Kim, J. Lee, S. Lee, S. W. Lee, C. S. Moon, Y. D. Oh, S. I. Pak, S. Sekmen, D. C. Son, Y. C. Yang, H. Kim, D. H. Moon, G. Oh, B. Francois, J. Goh, T. J. Kim, S. Cho, S. Choi, Y. Go, D. Gyun, S. Ha, B. Hong, Y. Jo, K. Lee, K. S. Lee, S. Lee, J. Lim, S. K. Park, Y. Roh, H. S. Kim, J. Almond, J. Kim, J. S. Kim, H. Lee, K. Lee, K. Nam, S. B. Oh, B. C. Radburn-Smith, S. h. Seo, U. K. Yang, H. D. Yoo, G. B. Yu, D. Jeon, H. Kim, J. H. Kim, J. S. H. Lee, I. C. Park, Y. Choi, C. Hwang, J. Lee, I. Yu, V. Dudenas, A. Juodagalvis, J. Vaitkus, Z. A. Ibrahim, M. A. B. Md Ali, F. Mohamad Idris, W. A. T. Wan Abdullah, M. N. Yusli, Z. Zolkapli, J. F. Benitez, A. Castaneda Hernandez, J. A. Murillo Quijada, H. Castilla-Valdez, E. De La Cruz-Burelo, M. C. Duran-Osuna, I. Heredia-De La Cruz, R. Lopez-Fernandez, J. Mejia Guisao, R. I. Rabadan-Trejo, M. Ramirez-Garcia, G. Ramirez-Sanchez, R. Reyes-Almanza, A. Sanchez-Hernandez, S. Carrillo Moreno, C. Oropeza Barrera, F. Vazquez Valencia, J. Eysermans, I. Pedraza, H. A. Salazar Ibarguen, C. Uribe Estrada, A. Morelos Pineda, D. Krofcheck, S. Bheesette, P. H. Butler, A. Ahmad, M. Ahmad, M. I. Asghar, Q. Hassan, H. R. Hoorani, W. A. Khan, A. Saddique, M. A. Shah, M. Shoaib, M. Waqas, H. Bialkowska, M. Bluj, B. Boimska, T. Frueboes, M. Górski, M. Kazana, M. Szleper, P. Traczyk, P. Zalewski, K. Bunkowski, A. Byszuk, K. Doroba, A. Kalinowski, M. Konecki, J. Krolikowski, M. Misiura, M. Olszewski, A. Pyskir, M. Walczak, M. Araujo, P. Bargassa, C. Beirão Da Cruz E Silva, A. Di Francesco, P. Faccioli, B. Galinhas, M. Gallinaro, J. Hollar, N. Leonardo, J. Seixas, G. Strong, O. Toldaiev, J. Varela, S. Afanasiev, P. Bunin, M. Gavrilenko, I. Golutvin, I. Gorbunov, A. Kamenev, V. Karjavine, A. Lanev, A. Malakhov, V. Matveev, P. Moisenz, V. Palichik, V. Perelygin, S. Shmatov, S. Shulha, N. Skatchkov, V. Smirnov, N. Voytishin, A. Zarubin, V. Golovtsov, Y. Ivanov, V. Kim, E. Kuznetsova, P. Levchenko, V. Murzin, V. Oreshkin, I. Smirnov, D. Sosnov, V. Sulimov, L. Uvarov, S. Vavilov, A. Vorobyev, Yu. Andreev, A. Dermenev, S. Gninenko, N. Golubev, A. Karneyeu, M. Kirsanov, N. Krasnikov, A. Pashenkov, A. Shabanov, D. Tlisov, A. Toropin, V. Epshteyn, V. Gavrilov, N. Lychkovskaya, V. Popov, I. Pozdnyakov, G. Safronov, A. Spiridonov, A. Stepennov, V. Stolin, M. Toms, E. Vlasov, A. Zhokin, T. Aushev, R. Chistov, M. Danilov, S. Polikarpov, E. Tarkovskii, V. Andreev, M. Azarkin, I. Dremin, M. Kirakosyan, A. Terkulov, A. Baskakov, A. Belyaev, E. Boos, M. Dubinin, L. Dudko, A. Ershov, A. Gribushin, V. Klyukhin, O. Kodolova, I. Lokhtin, I. Miagkov, S. Obraztsov, S. Petrushanko, V. Savrin, A. Snigirev, A. Barnyakov, V. Blinov, T. Dimova, L. Kardapoltsev, Y. Skovpen, I. Azhgirey, I. Bayshev, S. Bitioukov, V. Kachanov, A. Kalinin, D. Konstantinov, P. Mandrik, V. Petrov, R. Ryutin, S. Slabospitskii, A. Sobol, S. Troshin, N. Tyurin, A. Uzunian, A. Volkov, A. Babaev, S. Baidali, V. Okhotnikov, P. Adzic, P. Cirkovic, D. Devetak, M. Dordevic, J. Milosevic, J. Alcaraz Maestre, A. Álvarez Fernández, I. Bachiller, M. Barrio Luna, J. A. Brochero Cifuentes, M. Cerrada, N. Colino, B. De La Cruz, A. Delgado Peris, C. Fernandez Bedoya, J. P. Fernández Ramos, J. Flix, M. C. Fouz, O. Gonzalez Lopez, S. Goy Lopez, J. M. Hernandez, M. I. Josa, D. Moran, A. Pérez-Calero Yzquierdo, J. Puerta Pelayo, I. Redondo, L. Romero, S. Sánchez Navas, M. S. Soares, A. Triossi, C. Albajar, J. F. de Trocóniz, J. Cuevas, C. Erice, J. Fernandez Menendez, S. Folgueras, I. Gonzalez Caballero, J. R. González Fernández, E. Palencia Cortezon, V. Rodríguez Bouza, S. Sanchez Cruz, J. M. Vizan Garcia, I. J. Cabrillo, A. Calderon, B. Chazin Quero, J. Duarte Campderros, M. Fernandez, P. J. Fernández Manteca, A. García Alonso, J. Garcia-Ferrero, G. Gomez, A. Lopez Virto, J. Marco, C. Martinez Rivero, P. Martinez Ruiz del Arbol, F. Matorras, J. Piedra Gomez, C. Prieels, T. Rodrigo, A. Ruiz-Jimeno, L. Scodellaro, N. Trevisani, I. Vila, R. Vilar Cortabitarte, N. Wickramage, D. Abbaneo, B. Akgun, E. Auffray, G. Auzinger, P. Baillon, A. H. Ball, D. Barney, J. Bendavid, M. Bianco, A. Bocci, C. Botta, E. Brondolin, T. Camporesi, M. Cepeda, G. Cerminara, E. Chapon, Y. Chen, G. Cucciati, D. d’Enterria, A. Dabrowski, N. Daci, V. Daponte, A. David, A. De Roeck, N. Deelen, M. Dobson, M. Dünser, N. Dupont, A. Elliott-Peisert, P. Everaerts, F. Fallavollita, D. Fasanella, G. Franzoni, J. Fulcher, W. Funk, D. Gigi, A. Gilbert, K. Gill, F. Glege, M. Gruchala, M. Guilbaud, D. Gulhan, J. Hegeman, C. Heidegger, V. Innocente, A. Jafari, P. Janot, O. Karacheban, J. Kieseler, A. Kornmayer, M. Krammer, C. Lange, P. Lecoq, C. Lourenço, L. Malgeri, M. Mannelli, A. Massironi, F. Meijers, J. A. Merlin, S. Mersi, E. Meschi, P. Milenovic, F. Moortgat, M. Mulders, J. Ngadiuba, S. Nourbakhsh, S. Orfanelli, L. Orsini, F. Pantaleo, L. Pape, E. Perez, M. Peruzzi, A. Petrilli, G. Petrucciani, A. Pfeiffer, M. Pierini, F. M. Pitters, D. Rabady, A. Racz, T. Reis, M. Rovere, H. Sakulin, C. Schäfer, C. Schwick, M. Selvaggi, A. Sharma, P. Silva, P. Sphicas, A. Stakia, J. Steggemann, D. Treille, A. Tsirou, A. Vartak, V. Veckalns, M. Verzetti, W. D. Zeuner, L. Caminada, K. Deiters, W. Erdmann, R. Horisberger, Q. Ingram, H. C. Kaestli, D. Kotlinski, U. Langenegger, T. Rohe, S. A. Wiederkehr, M. Backhaus, L. Bäni, P. Berger, N. Chernyavskaya, G. Dissertori, M. Dittmar, M. Donegà, C. Dorfer, T. A. Gómez Espinosa, C. Grab, D. Hits, T. Klijnsma, W. Lustermann, R. A. Manzoni, M. Marionneau, M. T. Meinhard, F. Micheli, P. Musella, F. Nessi-Tedaldi, F. Pauss, G. Perrin, L. Perrozzi, S. Pigazzini, C. Reissel, D. Ruini, D. A. Sanz Becerra, M. Schönenberger, L. Shchutska, V. R. Tavolaro, K. Theofilatos, M. L. Vesterbacka Olsson, R. Wallny, D. H. Zhu, T. K. Aarrestad, C. Amsler, D. Brzhechko, M. F. Canelli, A. De Cosa, R. Del Burgo, S. Donato, C. Galloni, T. Hreus, B. Kilminster, S. Leontsinis, I. Neutelings, G. Rauco, P. Robmann, D. Salerno, K. Schweiger, C. Seitz, Y. Takahashi, A. Zucchetta, T. H. Doan, R. Khurana, C. M. Kuo, W. Lin, A. Pozdnyakov, S. S. Yu, P. Chang, Y. Chao, K. F. Chen, P. H. Chen, W.-S. Hou, Y. F. Liu, R.-S. Lu, E. Paganis, A. Psallidas, A. Steen, B. Asavapibhop, N. Srimanobhas, N. Suwonjandee, A. Bat, F. Boran, S. Cerci, S. Damarseckin, Z. S. Demiroglu, F. Dolek, C. Dozen, E. Eskut, G. Gokbulut, Y. Guler, E. Gurpinar, I. Hos, C. Isik, E. E. Kangal, O. Kara, A. Kayis Topaksu, U. Kiminsu, M. Oglakci, G. Onengut, K. Ozdemir, A. Polatoz, D. Sunar Cerci, B. Tali, U. G. Tok, S. Turkcapar, I. S. Zorbakir, C. Zorbilmez, B. Isildak, G. Karapinar, M. Yalvac, M. Zeyrek, I. O. Atakisi, E. Gülmez, M. Kaya, O. Kaya, S. Ozkorucuklu, S. Tekten, E. A. Yetkin, M. N. Agaras, A. Cakir, K. Cankocak, Y. Komurcu, S. Sen, B. Grynyov, L. Levchuk, F. Ball, J. J. Brooke, D. Burns, E. Clement, D. Cussans, O. Davignon, H. Flacher, J. Goldstein, G. P. Heath, H. F. Heath, L. Kreczko, D. M. Newbold, S. Paramesvaran, B. Penning, T. Sakuma, D. Smith, V. J. Smith, J. Taylor, A. Titterton, K. W. Bell, A. Belyaev, C. Brew, R. M. Brown, D. Cieri, D. J. A. Cockerill, J. A. Coughlan, K. Harder, S. Harper, J. Linacre, K. Manolopoulos, E. Olaiya, D. Petyt, C. H. Shepherd-Themistocleous, A. Thea, I. R. Tomalin, T. Williams, W. J. Womersley, R. Bainbridge, P. Bloch, J. Borg, S. Breeze, O. Buchmuller, A. Bundock, D. Colling, P. Dauncey, G. Davies, M. Della Negra, R. Di Maria, G. Hall, G. Iles, T. James, M. Komm, L. Lyons, A.-M. Magnan, S. Malik, A. Martelli, J. Nash, A. Nikitenko, V. Palladino, M. Pesaresi, D. M. Raymond, A. Richards, A. Rose, E. Scott, C. Seez, A. Shtipliyski, G. Singh, M. Stoye, T. Strebler, S. Summers, A. Tapper, K. Uchida, T. Virdee, N. Wardle, D. Winterbottom, S. C. Zenz, J. E. Cole, P. R. Hobson, A. Khan, P. Kyberd, C. K. Mackay, A. Morton, I. D. Reid, L. Teodorescu, S. Zahid, K. Call, J. Dittmann, K. Hatakeyama, H. Liu, C. Madrid, B. McMaster, N. Pastika, C. Smith, R. Bartek, A. Dominguez, A. Buccilli, S. I. Cooper, C. Henderson, P. Rumerio, C. West, D. Arcaro, T. Bose, D. Gastler, S. Girgis, D. Pinna, D. Rankin, C. Richardson, J. Rohlf, L. Sulak, D. Zou, G. Benelli, X. Coubez, D. Cutts, M. Hadley, J. Hakala, U. Heintz, J. M. Hogan, K. H. M. Kwok, E. Laird, G. Landsberg, J. Lee, Z. Mao, M. Narain, S. Sagir, R. Syarif, E. Usai, D. Yu, R. Band, C. Brainerd, R. Breedon, D. Burns, M. Calderon De La Barca Sanchez, M. Chertok, J. Conway, R. Conway, P. T. Cox, R. Erbacher, C. Flores, G. Funk, W. Ko, O. Kukral, R. Lander, M. Mulhearn, D. Pellett, J. Pilot, S. Shalhout, M. Shi, D. Stolp, D. Taylor, K. Tos, M. Tripathi, Z. Wang, F. Zhang, M. Bachtis, C. Bravo, R. Cousins, A. Dasgupta, A. Florent, J. Hauser, M. Ignatenko, N. Mccoll, S. Regnard, D. Saltzberg, C. Schnaible, V. Valuev, E. Bouvier, K. Burt, R. Clare, J. W. Gary, S. M. A. Ghiasi Shirazi, G. Hanson, G. Karapostoli, E. Kennedy, F. Lacroix, O. R. Long, M. Olmedo Negrete, M. I. Paneva, W. Si, L. Wang, H. Wei, S. Wimpenny, B. R. Yates, J. G. Branson, P. Chang, S. Cittolin, M. Derdzinski, R. Gerosa, D. Gilbert, B. Hashemi, A. Holzner, D. Klein, G. Kole, V. Krutelyov, J. Letts, M. Masciovecchio, D. Olivito, S. Padhi, M. Pieri, V. Sharma, S. Simon, M. Tadel, J. Wood, F. Würthwein, A. Yagil, G. Zevi Della Porta, N. Amin, R. Bhandari, C. Campagnari, M. Citron, V. Dutta, M. Franco Sevilla, L. Gouskos, R. Heller, J. Incandela, H. Mei, A. Ovcharova, H. Qu, J. Richman, D. Stuart, I. Suarez, S. Wang, J. Yoo, D. Anderson, A. Bornheim, J. M. Lawhorn, N. Lu, H. B. Newman, T. Q. Nguyen, J. Pata, M. Spiropulu, J. R. Vlimant, R. Wilkinson, S. Xie, Z. Zhang, R. Y. Zhu, M. B. Andrews, T. Ferguson, T. Mudholkar, M. Paulini, M. Sun, I. Vorobiev, M. Weinberg, J. P. Cumalat, W. T. Ford, F. Jensen, A. Johnson, E. MacDonald, T. Mulholland, R. Patel, A. Perloff, K. Stenson, K. A. Ulmer, S. R. Wagner, J. Alexander, J. Chaves, Y. Cheng, J. Chu, A. Datta, K. Mcdermott, N. Mirman, J. R. Patterson, D. Quach, A. Rinkevicius, A. Ryd, L. Skinnari, L. Soffi, S. M. Tan, Z. Tao, J. Thom, J. Tucker, P. Wittich, M. Zientek, S. Abdullin, M. Albrow, M. Alyari, G. Apollinari, A. Apresyan, A. Apyan, S. Banerjee, L. A. T. Bauerdick, A. Beretvas, J. Berryhill, P. C. Bhat, K. Burkett, J. N. Butler, A. Canepa, G. B. Cerati, H. W. K. Cheung, F. Chlebana, M. Cremonesi, J. Duarte, V. D. Elvira, J. Freeman, Z. Gecse, E. Gottschalk, L. Gray, D. Green, S. Grünendahl, O. Gutsche, J. Hanlon, R. M. Harris, S. Hasegawa, J. Hirschauer, Z. Hu, B. Jayatilaka, S. Jindariani, M. Johnson, U. Joshi, B. Klima, M. J. Kortelainen, B. Kreis, S. Lammel, D. Lincoln, R. Lipton, M. Liu, T. Liu, J. Lykken, K. Maeshima, J. M. Marraffino, D. Mason, P. McBride, P. Merkel, S. Mrenna, S. Nahn, V. O’Dell, K. Pedro, C. Pena, O. Prokofyev, G. Rakness, F. Ravera, A. Reinsvold, L. Ristori, A. Savoy-Navarro, B. Schneider, E. Sexton-Kennedy, A. Soha, W. J. Spalding, L. Spiegel, S. Stoynev, J. Strait, N. Strobbe, L. Taylor, S. Tkaczyk, N. V. Tran, L. Uplegger, E. W. Vaandering, C. Vernieri, M. Verzocchi, R. Vidal, M. Wang, H. A. Weber, A. Whitbeck, D. Acosta, P. Avery, P. Bortignon, D. Bourilkov, A. Brinkerhoff, L. Cadamuro, A. Carnes, D. Curry, R. D. Field, S. V. Gleyzer, B. M. Joshi, J. Konigsberg, A. Korytov, K. H. Lo, P. Ma, K. Matchev, G. Mitselmakher, D. Rosenzweig, K. Shi, D. Sperka, J. Wang, S. Wang, X. Zuo, Y. R. Joshi, S. Linn, A. Ackert, T. Adams, A. Askew, S. Hagopian, V. Hagopian, K. F. Johnson, T. Kolberg, G. Martinez, T. Perry, H. Prosper, A. Saha, C. Schiber, R. Yohay, M. M. Baarmand, V. Bhopatkar, S. Colafranceschi, M. Hohlmann, D. Noonan, M. Rahmani, T. Roy, M. Saunders, F. Yumiceva, M. R. Adams, L. Apanasevich, D. Berry, R. R. Betts, R. Cavanaugh, X. Chen, S. Dittmer, O. Evdokimov, C. E. Gerber, D. A. Hangal, D. J. Hofman, K. Jung, J. Kamin, C. Mills, M. B. Tonjes, N. Varelas, H. Wang, X. Wang, Z. Wu, J. Zhang, M. Alhusseini, B. Bilki, W. Clarida, K. Dilsiz, S. Durgut, R. P. Gandrajula, M. Haytmyradov, V. Khristenko, J.-P. Merlo, A. Mestvirishvili, A. Moeller, J. Nachtman, H. Ogul, Y. Onel, F. Ozok, A. Penzo, C. Snyder, E. Tiras, J. Wetzel, B. Blumenfeld, A. Cocoros, N. Eminizer, D. Fehling, L. Feng, A. V. Gritsan, W. T. Hung, P. Maksimovic, J. Roskes, U. Sarica, M. Swartz, M. Xiao, A. Al-bataineh, P. Baringer, A. Bean, S. Boren, J. Bowen, A. Bylinkin, J. Castle, S. Khalil, A. Kropivnitskaya, D. Majumder, W. Mcbrayer, M. Murray, C. Rogan, S. Sanders, E. Schmitz, J. D. Tapia Takaki, Q. Wang, S. Duric, A. Ivanov, K. Kaadze, D. Kim, Y. Maravin, D. R. Mendis, T. Mitchell, A. Modak, A. Mohammadi, F. Rebassoo, D. Wright, A. Baden, O. Baron, A. Belloni, S. C. Eno, Y. Feng, C. Ferraioli, N. J. Hadley, S. Jabeen, G. Y. Jeng, R. G. Kellogg, J. Kunkle, A. C. Mignerey, S. Nabili, F. Ricci-Tam, M. Seidel, Y. H. Shin, A. Skuja, S. C. Tonwar, K. Wong, D. Abercrombie, B. Allen, V. Azzolini, A. Baty, G. Bauer, R. Bi, S. Brandt, W. Busza, I. A. Cali, M. D’Alfonso, Z. Demiragli, G. Gomez Ceballos, M. Goncharov, P. Harris, D. Hsu, M. Hu, Y. Iiyama, G. M. Innocenti, M. Klute, D. Kovalskyi, Y.-J. Lee, P. D. Luckey, B. Maier, A. C. Marini, C. Mcginn, C. Mironov, S. Narayanan, X. Niu, C. Paus, C. Roland, G. Roland, Z. Shi, G. S. F. Stephans, K. Sumorok, K. Tatar, D. Velicanu, J. Wang, T. W. Wang, B. Wyslouch, A. C. Benvenuti, R. M. Chatterjee, A. Evans, P. Hansen, J. Hiltbrand, Sh. Jain, S. Kalafut, M. Krohn, Y. Kubota, Z. Lesko, J. Mans, N. Ruckstuhl, R. Rusack, M. A. Wadud, J. G. Acosta, S. Oliveros, E. Avdeeva, K. Bloom, D. R. Claes, C. Fangmeier, F. Golf, R. Gonzalez Suarez, R. Kamalieddin, I. Kravchenko, J. Monroy, J. E. Siado, G. R. Snow, B. Stieger, A. Godshalk, C. Harrington, I. Iashvili, A. Kharchilava, C. Mclean, D. Nguyen, A. Parker, S. Rappoccio, B. Roozbahani, G. Alverson, E. Barberis, C. Freer, Y. Haddad, A. Hortiangtham, G. Madigan, D. M. Morse, T. Orimoto, A. Tishelman-charny, T. Wamorkar, B. Wang, A. Wisecarver, D. Wood, S. Bhattacharya, J. Bueghly, O. Charaf, T. Gunter, K. A. Hahn, N. Odell, M. H. Schmitt, K. Sung, M. Trovato, M. Velasco, R. Bucci, N. Dev, M. Hildreth, K. Hurtado Anampa, C. Jessop, D. J. Karmgard, K. Lannon, W. Li, N. Loukas, N. Marinelli, F. Meng, C. Mueller, Y. Musienko, M. Planer, R. Ruchti, P. Siddireddy, G. Smith, S. Taroni, M. Wayne, A. Wightman, M. Wolf, A. Woodard, J. Alimena, L. Antonelli, B. Bylsma, L. S. Durkin, S. Flowers, B. Francis, C. Hill, W. Ji, T. Y. Ling, W. Luo, B. L. Winer, S. Cooperstein, P. Elmer, J. Hardenbrook, N. Haubrich, S. Higginbotham, A. Kalogeropoulos, S. Kwan, D. Lange, M. T. Lucchini, J. Luo, D. Marlow, K. Mei, I. Ojalvo, J. Olsen, C. Palmer, P. Piroué, J. Salfeld-Nebgen, D. Stickland, C. Tully, S. Malik, S. Norberg, A. Barker, V. E. Barnes, S. Das, L. Gutay, M. Jones, A. W. Jung, A. Khatiwada, B. Mahakud, D. H. Miller, N. Neumeister, C. C. Peng, S. Piperov, H. Qiu, J. F. Schulte, J. Sun, F. Wang, R. Xiao, W. Xie, T. Cheng, J. Dolen, N. Parashar, Z. Chen, K. M. Ecklund, S. Freed, F. J. M. Geurts, M. Kilpatrick, Arun Kumar, W. Li, B. P. Padley, R. Redjimi, J. Roberts, J. Rorie, W. Shi, Z. Tu, A. Zhang, A. Bodek, P. de Barbaro, R. Demina, Y. t. Duh, J. L. Dulemba, C. Fallon, T. Ferbel, M. Galanti, A. Garcia-Bellido, J. Han, O. Hindrichs, A. Khukhunaishvili, E. Ranken, P. Tan, R. Taus, B. Chiarito, J. P. Chou, Y. Gershtein, E. Halkiadakis, A. Hart, M. Heindl, E. Hughes, S. Kaplan, R. Kunnawalkam Elayavalli, S. Kyriacou, I. Laflotte, A. Lath, R. Montalvo, K. Nash, M. Osherson, H. Saka, S. Salur, S. Schnetzer, D. Sheffield, S. Somalwar, R. Stone, S. Thomas, P. Thomassen, A. G. Delannoy, J. Heideman, G. Riley, S. Spanier, O. Bouhali, A. Celik, M. Dalchenko, M. De Mattia, A. Delgado, S. Dildick, R. Eusebi, J. Gilmore, T. Huang, T. Kamon, S. Luo, D. Marley, R. Mueller, D. Overton, L. Perniè, D. Rathjens, A. Safonov, N. Akchurin, J. Damgov, F. De Guio, P. R. Dudero, S. Kunori, K. Lamichhane, S. W. Lee, T. Mengke, S. Muthumuni, T. Peltola, S. Undleeb, I. Volobouev, Z. Wang, S. Greene, A. Gurrola, R. Janjam, W. Johns, C. Maguire, A. Melo, H. Ni, K. Padeken, F. Romeo, J. D. Ruiz Alvarez, P. Sheldon, S. Tuo, J. Velkovska, M. Verweij, Q. Xu, M. W. Arenton, P. Barria, B. Cox, R. Hirosky, M. Joyce, A. Ledovskoy, H. Li, C. Neu, T. Sinthuprasith, Y. Wang, E. Wolfe, F. Xia, R. Harr, P. E. Karchin, N. Poudyal, J. Sturdy, P. Thapa, S. Zaleski, J. Buchanan, C. Caillol, D. Carlsmith, S. Dasu, I. De Bruyn, L. Dodd, B. Gomber, M. Grothe, M. Herndon, A. Hervé, U. Hussain, P. Klabbers, A. Lanaro, K. Long, R. Loveless, T. Ruggles, A. Savin, V. Sharma, N. Smith, W. H. Smith, N. Woods, The CMS Collaboration

**Affiliations:** 1 Yerevan Physics Institute, Yerevan, Armenia; 2 Institut für Hochenergiephysik, Wien, Austria; 3 Institute for Nuclear Problems, Minsk, Belarus; 4 Universiteit Antwerpen, Antwerpen, Belgium; 5 Vrije Universiteit Brussel, Brussel, Belgium; 6 Université Libre de Bruxelles, Bruxelles, Belgium; 7 Ghent University, Ghent, Belgium; 8 Université Catholique de Louvain, Louvain-la-Neuve, Belgium; 9 Centro Brasileiro de Pesquisas Fisicas, Rio de Janeiro, Brazil; 10 Universidade do Estado do Rio de Janeiro, Rio de Janeiro, Brazil; 11 Universidade Estadual Paulista, Universidade Federal do ABC, São Paulo, Brazil; 12 Institute for Nuclear Research and Nuclear Energy, Bulgarian Academy of Sciences, Sofia, Bulgaria; 13 University of Sofia, Sofia, Bulgaria; 14 Beihang University, Beijing, China; 15 Institute of High Energy Physics, Beijing, China; 16 State Key Laboratory of Nuclear Physics and Technology, Peking University, Beijing, China; 17 Tsinghua University, Beijing, China; 18 Universidad de Los Andes, Bogota, Colombia; 19 Faculty of Electrical Engineering, Mechanical Engineering and Naval Architecture, University of Split, Split, Croatia; 20 Faculty of Science, University of Split, Split, Croatia; 21 Institute Rudjer Boskovic, Zagreb, Croatia; 22 University of Cyprus, Nicosia, Cyprus; 23 Charles University, Prague, Czech Republic; 24 Escuela Politecnica Nacional, Quito, Ecuador; 25 Universidad San Francisco de Quito, Quito, Ecuador; 26 Academy of Scientific Research and Technology of the Arab Republic of Egypt, Egyptian Network of High Energy Physics, Cairo, Egypt; 27 National Institute of Chemical Physics and Biophysics, Tallinn, Estonia; 28 Department of Physics, University of Helsinki, Helsinki, Finland; 29 Helsinki Institute of Physics, Helsinki, Finland; 30 Lappeenranta University of Technology, Lappeenranta, Finland; 31 IRFU, CEA, Université Paris-Saclay, Gif-sur-Yvette, France; 32 Laboratoire Leprince-Ringuet, Ecole polytechnique, CNRS/IN2P3, Université Paris-Saclay, Palaiseau, France; 33 Université de Strasbourg, CNRS, IPHC UMR 7178, Strasbourg, France; 34 Centre de Calcul de l’Institut National de Physique Nucleaire et de Physique des Particules, CNRS/IN2P3, Villeurbanne, France; 35 CNRS-IN2P3, Institut de Physique Nucléaire de Lyon, Université de Lyon, Université Claude Bernard Lyon 1, Villeurbanne, France; 36 Georgian Technical University, Tbilisi, Georgia; 37 Tbilisi State University, Tbilisi, Georgia; 38 RWTH Aachen University, I. Physikalisches Institut, Aachen, Germany; 39 RWTH Aachen University, III. Physikalisches Institut A, Aachen, Germany; 40 RWTH Aachen University, III. Physikalisches Institut B, Aachen, Germany; 41 Deutsches Elektronen-Synchrotron, Hamburg, Germany; 42 University of Hamburg, Hamburg, Germany; 43 Karlsruher Institut fuer Technologie, Karlsruhe, Germany; 44 Institute of Nuclear and Particle Physics (INPP), NCSR Demokritos, Aghia Paraskevi, Greece; 45 National and Kapodistrian University of Athens, Athens, Greece; 46 National Technical University of Athens, Athens, Greece; 47 University of Ioánnina, Ioánnina, Greece; 48 MTA-ELTE Lendület CMS Particle and Nuclear Physics Group, Eötvös Loránd University, Budapest, Hungary; 49 Wigner Research Centre for Physics, Budapest, Hungary; 50 Institute of Nuclear Research ATOMKI, Debrecen, Hungary; 51 Institute of Physics, University of Debrecen, Debrecen, Hungary; 52 Indian Institute of Science (IISc), Bangalore, India; 53 National Institute of Science Education and Research, HBNI, Bhubaneswar, India; 54 Panjab University, Chandigarh, India; 55 University of Delhi, Delhi, India; 56 Saha Institute of Nuclear Physics, HBNI, Kolkata, India; 57 Indian Institute of Technology Madras, Madras, India; 58 Bhabha Atomic Research Centre, Mumbai, India; 59 Tata Institute of Fundamental Research-A, Mumbai, India; 60 Tata Institute of Fundamental Research-B, Mumbai, India; 61 Indian Institute of Science Education and Research (IISER), Pune, India; 62 Institute for Research in Fundamental Sciences (IPM), Tehran, Iran; 63 University College Dublin, Dublin, Ireland; 64 INFN Sezione di Bari, Università di Bari, Politecnico di Bari, Bari, Italy; 65 INFN Sezione di Bologna, Università di Bologna, Bologna, Italy; 66 INFN Sezione di Catania, Università di Catania, Catania, Italy; 67 INFN Sezione di Firenze, Università di Firenze, Firenze, Italy; 68 INFN Laboratori Nazionali di Frascati, Frascati, Italy; 69 INFN Sezione di Genova, Università di Genova, Genova, Italy; 70 INFN Sezione di Milano-Bicocca, Università di Milano-Bicocca, Milano, Italy; 71 INFN Sezione di Napoli, Università di Napoli ’Federico II’ , Napoli, Italy, Università della Basilicata, Potenza, Italy, Università G. Marconi, Roma, Italy; 72 INFN Sezione di Padova, Università di Padova, Padova, Italy, Università di Trento, Trento, Italy; 73 INFN Sezione di Pavia, Università di Pavia, Pavia, Italy; 74 INFN Sezione di Perugia, Università di Perugia, Perugia, Italy; 75 INFN Sezione di Pisa, Università di Pisa, Scuola Normale Superiore di Pisa, Pisa, Italy; 76 INFN Sezione di Roma, Sapienza Università di Roma, Rome, Italy; 77 INFN Sezione di Torino, Università di Torino, Torino, Italy, Università del Piemonte Orientale, Novara, Italy; 78 INFN Sezione di Trieste, Università di Trieste, Trieste, Italy; 79 Kyungpook National University, Daegu, Korea; 80 Chonnam National University, Institute for Universe and Elementary Particles, Kwangju, Korea; 81 Hanyang University, Seoul, Korea; 82 Korea University, Seoul, Korea; 83 Sejong University, Seoul, Korea; 84 Seoul National University, Seoul, Korea; 85 University of Seoul, Seoul, Korea; 86 Sungkyunkwan University, Suwon, Korea; 87 Vilnius University, Vilnius, Lithuania; 88 National Centre for Particle Physics, Universiti Malaya, Kuala Lumpur, Malaysia; 89 Universidad de Sonora (UNISON), Hermosillo, Mexico; 90 Centro de Investigacion y de Estudios Avanzados del IPN, Mexico City, Mexico; 91 Universidad Iberoamericana, Mexico City, Mexico; 92 Benemerita Universidad Autonoma de Puebla, Puebla, Mexico; 93 Universidad Autónoma de San Luis Potosí, San Luis Potosí, Mexico; 94 University of Auckland, Auckland, New Zealand; 95 University of Canterbury, Christchurch, New Zealand; 96 National Centre for Physics, Quaid-I-Azam University, Islamabad, Pakistan; 97 National Centre for Nuclear Research, Swierk, Poland; 98 Institute of Experimental Physics, Faculty of Physics, University of Warsaw, Warsaw, Poland; 99 Laboratório de Instrumentação e Física Experimental de Partículas, Lisboa, Portugal; 100 Joint Institute for Nuclear Research, Dubna, Russia; 101 Petersburg Nuclear Physics Institute, Gatchina (St. Petersburg), Russia; 102 Institute for Nuclear Research, Moscow, Russia; 103 Institute for Theoretical and Experimental Physics, Moscow, Russia; 104 Moscow Institute of Physics and Technology, Moscow, Russia; 105 National Research Nuclear University ’Moscow Engineering Physics Institute’ (MEPhI), Moscow, Russia; 106 P.N. Lebedev Physical Institute, Moscow, Russia; 107 Skobeltsyn Institute of Nuclear Physics, Lomonosov Moscow State University, Moscow, Russia; 108 Novosibirsk State University (NSU), Novosibirsk, Russia; 109 Institute for High Energy Physics of National Research Centre ’Kurchatov Institute’, Protvino, Russia; 110 National Research Tomsk Polytechnic University, Tomsk, Russia; 111 Faculty of Physics and VINCA Institute of Nuclear Sciences, University of Belgrade, Belgrade, Serbia; 112 Centro de Investigaciones Energéticas Medioambientales y Tecnológicas (CIEMAT), Madrid, Spain; 113 Universidad Autónoma de Madrid, Madrid, Spain; 114 Universidad de Oviedo, Oviedo, Spain; 115 Instituto de Física de Cantabria (IFCA), CSIC-Universidad de Cantabria, Santander, Spain; 116 Department of Physics, University of Ruhuna, Matara, Sri Lanka; 117 CERN, European Organization for Nuclear Research, Geneva, Switzerland; 118 Paul Scherrer Institut, Villigen, Switzerland; 119 ETH Zurich, Institute for Particle Physics and Astrophysics (IPA), Zurich, Switzerland; 120 Universität Zürich, Zurich, Switzerland; 121 National Central University, Chung-Li, Taiwan; 122 National Taiwan University (NTU), Taipei, Taiwan; 123 Chulalongkorn University, Faculty of Science, Department of Physics, Bangkok, Thailand; 124 Çukurova University, Physics Department, Science and Art Faculty, Adana, Turkey; 125 Middle East Technical University, Physics Department, Ankara, Turkey; 126 Bogazici University, Istanbul, Turkey; 127 Istanbul Technical University, Istanbul, Turkey; 128 Institute for Scintillation Materials of National Academy of Science of Ukraine, Kharkov, Ukraine; 129 National Scientific Center, Kharkov Institute of Physics and Technology, Kharkov, Ukraine; 130 University of Bristol, Bristol, UK; 131 Rutherford Appleton Laboratory, Didcot, UK; 132 Imperial College, London, UK; 133 Brunel University, Uxbridge, UK; 134 Baylor University, Waco, USA; 135 Catholic University of America, Washington, DC USA; 136 The University of Alabama, Tuscaloosa, USA; 137 Boston University, Boston, USA; 138 Brown University, Providence, USA; 139 University of California, Davis, Davis USA; 140 University of California, Los Angeles, USA; 141 University of California, Riverside, Riverside, USA; 142 University of California, San Diego, La Jolla, USA; 143 Department of Physics, University of California, Santa Barbara, Santa Barbara, USA; 144 California Institute of Technology, Pasadena, USA; 145 Carnegie Mellon University, Pittsburgh, USA; 146 University of Colorado Boulder, Boulder, USA; 147 Cornell University, Ithaca, USA; 148 Fermi National Accelerator Laboratory, Batavia, USA; 149 University of Florida, Gainesville, USA; 150 Florida International University, Miami, USA; 151 Florida State University, Tallahassee, USA; 152 Florida Institute of Technology, Melbourne, USA; 153 University of Illinois at Chicago (UIC), Chicago, USA; 154 The University of Iowa, Iowa City, USA; 155 Johns Hopkins University, Baltimore, USA; 156 The University of Kansas, Lawrence, USA; 157 Kansas State University, Manhattan, USA; 158 Lawrence Livermore National Laboratory, Livermore, USA; 159 University of Maryland, College Park, USA; 160 Massachusetts Institute of Technology, Cambridge, USA; 161 University of Minnesota, Minneapolis, USA; 162 University of Mississippi, Oxford, USA; 163 University of Nebraska-Lincoln, Lincoln, USA; 164 State University of New York at Buffalo, Buffalo, USA; 165 Northeastern University, Boston, USA; 166 Northwestern University, Evanston, USA; 167 University of Notre Dame, Notre Dame, USA; 168 The Ohio State University, Columbus, USA; 169 Princeton University, Princeton, USA; 170 University of Puerto Rico, Mayaguez, USA; 171 Purdue University, West Lafayette, USA; 172 Purdue University Northwest, Hammond, USA; 173 Rice University, Houston, USA; 174 University of Rochester, Rochester, USA; 175 Rutgers, The State University of New Jersey, Piscataway, USA; 176 University of Tennessee, Knoxville, USA; 177 Texas A & M University, College Station, USA; 178 Texas Tech University, Lubbock, USA; 179 Vanderbilt University, Nashville, USA; 180 University of Virginia, Charlottesville, USA; 181 Wayne State University, Detroit, USA; 182 University of Wisconsin, Madison, Madison, WI USA

## Abstract

New sets of CMS underlying-event parameters (“tunes”) are presented for the pythia8 event generator. These tunes use the NNPDF3.1 parton distribution functions (PDFs) at leading (LO), next-to-leading (NLO), or next-to-next-to-leading (NNLO) orders in perturbative quantum chromodynamics, and the strong coupling evolution at LO or NLO. Measurements of charged-particle multiplicity and transverse momentum densities at various hadron collision energies are fit simultaneously to determine the parameters of the tunes. Comparisons of the predictions of the new tunes are provided for observables sensitive to the event shapes at LEP, global underlying event, soft multiparton interactions, and double-parton scattering contributions. In addition, comparisons are made for observables measured in various specific processes, such as multijet, Drell–Yan, and top quark-antiquark pair production including jet substructure observables. The simulation of the underlying event provided by the new tunes is interfaced to a higher-order matrix-element calculation. For the first time, predictions from pythia8 obtained with tunes based on NLO or NNLO PDFs are shown to reliably describe minimum-bias and underlying-event data with a similar level of agreement to predictions from tunes using LO PDF sets.

## Introduction

Monte Carlo (MC) simulation codes describe hadron-hadron collisions with models based on several components. The hard scattering component of the event consists of particles from the hadronization of partons whose kinematics are predicted using perturbative matrix elements (MEs), along with partons from initial-state radiation (ISR) and final-state radiation (FSR) that are simulated using a showering algorithm. The underlying event (UE) consists of the beam-beam remnants (BBR) and the particles that arise from multiple-parton interactions (MPI). The BBR are what remains after a parton is scattered out of each of the two initial beam hadrons. The MPI are additional soft or semi-hard parton–parton scatterings that occur within the same hadron–hadron collision. Generally, observables sensitive to the UE also receive contributions from the hard-scattering components. Accurately describing observables that are sensitive to the UE not only requires a good description of BBR and MPI, but also a good modeling of hadronization, ISR, and FSR. Standard MC event generators, such as pythia8 [1], herwig [2, 3], and sherpa [4] have adjustable parameters to control the behavior of their event modeling. A set of these parameters, which has been adjusted to better fit some aspects of the data, is referred to as a tune.

In a previous study [5], we presented several pythia8 and herwig++ UE tunes constructed for a center-of-mass energy $\sqrt{s}$ lower than 13$\,\text {TeV}$. The CMS pythia8 tune CUETP8M1 is based on the Monash tune [6], both using the NNPDF2.3LO parton distribution function (PDF) set [7]. The CMS pythia8 tune CUETP8S1-CTEQ6L1 is based instead on the tune 4C [8]. Both tunes CUETP8M1 and CUETP8S1-CTEQ6L1 were constructed by fitting the CDF UE data at $\sqrt{s}=900\,\text {GeV}$ and 1.96$\,\text {TeV}$ [9] together with CMS UE data at $\sqrt{s}=7\,\text {TeV}$ [10]. A similar procedure was used for the determination of the herwig++ tune (CUETHppS1) with the CTEQ6L1 PDF set [11]. A collection of previously published tunes is documented in [6, 8, 12, 12–15].

In this paper, a new set of tunes for the UE simulation in the pythia8 (version 8.226) event generator is obtained by fitting various measurements sensitive to soft and semi-hard MPI at different hadron collision energies [9, 10], including data from $\sqrt{s}=13\,\text {TeV}$ [16]. These tunes are constructed with the leading order (LO), next-to-leading order (NLO), and next-to-next-to-leading order (NNLO) versions of the NNPDF3.1 PDF set [17] for the simulation of all UE components. Typically, the values of strong coupling used for the simulation of the hard scattering are chosen consistent with the order of the PDF set used.

The new tunes are obtained by fitting CDF UE data at $\sqrt{s}=1.96\,\text {TeV}$ [9], together with CMS UE data at $\sqrt{s}=7\,\text {TeV}$ [10] and at 13$\,\text {TeV}$ [16, 18]. For the first time, we show that predictions obtained with tunes based on higher-order PDF sets are able to give a reliable description of minimum-bias (MB) and UE measurements with a similar level of agreement to predictions from tunes using LO PDF sets. We also compare the predictions for multijet, Drell–Yan, and top-antiquark ($\mathrm{t}\overline{\mathrm{t}}$) processes from pythia8 with new tunes in ME-parton shower (PS) merged configurations.

In Sect. 2 we describe observables that are sensitive to MB and UE: diffractive processes [19], where one or both protons remain intact after the collision; and double-parton scattering (DPS), where two hard scatterings occur within the same collision. In Sect. 3, we compare the tunes that were constructed before the data at $\sqrt{s}=13\,\text {TeV}$ were available (“Pre-13$\,\text {TeV}$ ” tunes) with UE data measured at 13$\,\text {TeV}$. Section 4 is dedicated to a general discussion of the choice of PDF sets and strong coupling values for the UE simulation. In Sect. 5 we describe the new tunes. Section 6 shows the validation of the new CMS pythia8 tunes for multijet, Drell–Yan, $\mathrm{t}\overline{\mathrm{t}}$, and DPS processes. Section 7 is the summary and conclusions.

## Observables for characterizing minimum bias, underlying event, and double-parton scattering

Minimum bias is a generic term that refers to inelastic events that are collected with a loose event selection that has the smallest bias possible. The MB observables are constructed from data with little or no additional selection requirements. The majority of MB collisions are soft, with a typical transverse momentum scale $p_{\mathrm {T}} \lesssim 2\,\text {GeV}$. The UE is defined as the activity that is not associated with the particles originating from the hard scattering of two partons and is generally studied in events that contain a hard scattering with $p_{\mathrm {T}} \gtrsim 2\,\text {GeV}$. The main contribution to the UE comes from color exchanges between the beam partons and is modeled in terms of MPI, BBR, and color reconnection (CR). The MB and UE observables have quite different kinematic properties because they are affected by different mixtures of hard and soft scattering processes.

**Fig. 1:**
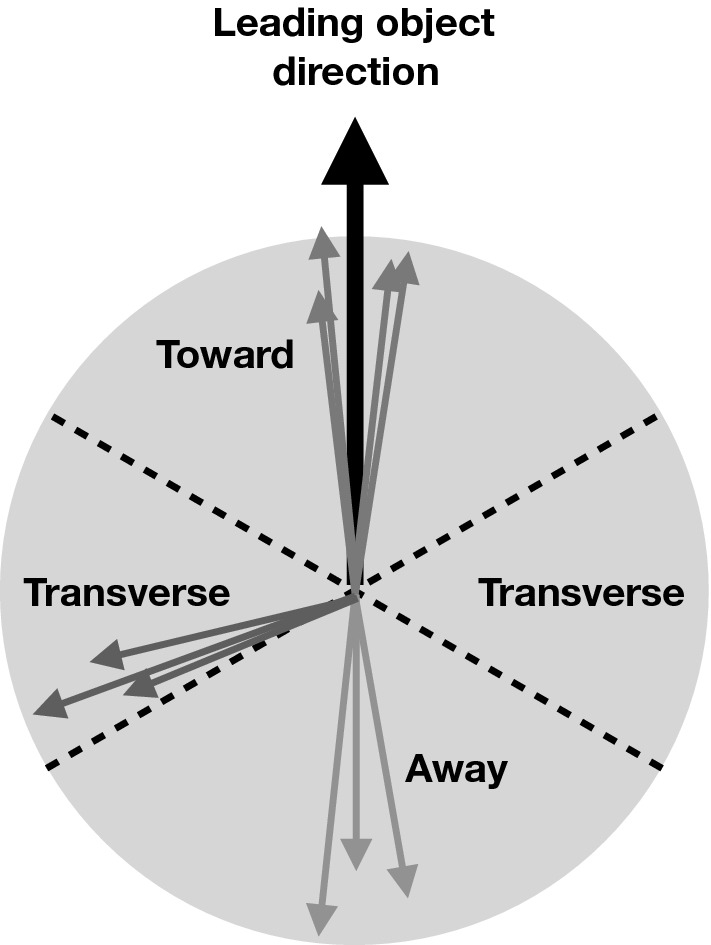
Illustration of several $\phi$ regions relative to the leading object that are sensitive to the underlying event. See the text for the details on the definitions of the regions

As illustrated in Fig. 1, one can use the topological structure of a typical hard hadron-hadron collision to study the UE experimentally. On an event-by-event basis, a leading object is used to define regions of $\eta \text {-}\phi$ space that are sensitive to the modeling of the UE, where $\eta$ is the pseudorapidity and $\phi$ is the azimuthal scattering angle defined in the *xy* plane. The azimuthal separation between charged particles and the leading object, $\varDelta \phi \ = \phi - \phi _{\text {max}}$, is used to define the UE-sensitive regions. Here $\phi _{\text {max}}$ is the azimuth of the leading object and $\phi$ is the azimuth angle of an outgoing charged particle. The regions are labelled as ‘toward’ ($|\varDelta \phi |\le 60^{\circ }$), ‘away’ ($|\varDelta \phi |>120^{\circ }$), and ‘transverse’ ($60^{\circ }<|\varDelta \phi |\le 120^{\circ }$). The transverse region can further be separated into transMAX and transMIN. On an event-by-event basis, transMAX (transMIN) is defined as the transverse region having the maximum (minimum) of either the number of charged particles, or scalar $p_{\mathrm {T}}$ sum of charged particles ($p_{\mathrm {T}} ^\text {sum}$), depending on the quantity under study.

Published UE studies used the charged-particle jet with the largest $p_{\mathrm {T}}$ [16], the dilepton system in DY [20, 21], or $\mathrm{t}\overline{\mathrm{t}}$ [22] events as the leading (i.e., the highest $p_{\mathrm {T}}$) objects. The tunes from CDF and CMS data [9, 10] made use of the charged particle with the largest $p_{\mathrm {T}}$ ($p_{\mathrm {T}} ^\text {max}$) as the “leading object”, and use only charged particles with $p_{\mathrm {T}} > 0.5 \,\text {GeV}$ and $|\eta | < 0.8$ to characterize the UE. The toward region contains the leading object, and the away region is expected to include the object recoiling against the leading one. Most of the UE contributions, i.e., PS and MPI, are contained in the two transverse regions. For events with multiple ISR or FSR emissions, transMAX often contains a third hard jet, while both transMAX and transMIN receive contributions from the MPI and BBR components. Typically, the transMIN observables are more sensitive to the MPI and BBR components of the UE.

Observables sensitive to UE contributions are the charged-particle multiplicity and the charged-particle scalar-$p_{\mathrm {T}}$ sum densities in the $\eta \text {-}\phi$ space, measured in transMIN and transMAX. The tunes that are constructed by fitting such UE-sensitive observables are referred to as “UE tunes”.

The pythia8 MC event generator also simulates single-diffractive (SD) dissociation, double-diffractive (DD) dissociation, central-diffractive (CD), and nondiffractive (ND) processes [23], which contribute to the inelastic cross section in hadron-hadron collisions. In SD, CD, and DD events, one or both of the beam particles are excited into color singlet states, which then decay. The SD and DD processes correspond to color singlet exchanges between the beam hadrons, while CD corresponds to double color singlet exchange with a diffractive system produced centrally. For ND processes, color exchanges occur, the outgoing remnants are no longer color singlets, and a multitude of particles is produced. All processes except SD are defined as nonSD (NSD) processes. An NSD-enhanced sample is required to have an energy deposit in both the backward ($-5< \eta < -3$) and the forward ($3< \eta < 5$) regions of the detector. The details of the selection for different types of diffractive events can be found in Ref. [24].

Generally, MC models such as pythia8 regularize the contributions of the primary hard-scattering processes and MPI to the differential cross section by using a threshold parameter $p_{\mathrm {T}} ^0$. The primary hard-scattering processes and the MPI are regularized in the same way with this parameter. This threshold is expected to have a dependence on the center-of-mass energy of the hadron-hadron collision, $\sqrt{s}$. The threshold at a reference center-of-mass energy $\sqrt{s}=7\,\text {TeV}$ is called pT0Ref. In pythia8 the energy dependence is parameterized using a power law function with a reference energy parameter $s_0$ and an exponent $\epsilon$. At a given center-of-mass energy, the amount of MPI depends on the threshold $p_{\mathrm {T}} ^0$, the PDF, and the overlap of the matter distributions of the two colliding hadrons. Smaller values of $p_{\mathrm {T}} ^0$ result in larger MPI contributions because of a higher MPI cross section. Each MPI adds colored partons to the final state, creating a dense net of color lines that spatially overlap with the fields produced by the partons of the hard scattering and with each other. All the generated color lines may connect to each other according to the CR model.

Since pythia8 regularizes both the cross section for MPI and the cross section of collisions with low-$p_{\mathrm {T}}$ exchange using the $p_{\mathrm {T}} ^0$ parameter, one can model the overall ND cross section by letting the $p_{\mathrm {T}}$  of the primary hard scattering become small. In this simple approach, the UE in a hard-scattering process is related to MB collisions. At the same center-of-mass energy, the activity in the UE of a hard-scattering process is greater than that of an average MB collision. In pythia8, this is caused by the higher MPI activity in hard-scattering processes compared to a typical MB collision. By demanding a hard scattering, one forces the collision to be more central, i.e., with a small impact parameter between the protons, and this increases the probability of MPI. For MB collisions, peripheral collisions, where the impact parameter between the two colliding protons is large, are most common.

Typically MPI interactions contain particles with substantially lower $p_{\mathrm {T}}$ (“softer”). However, occasionally two hard 2-to-2 parton scatterings can occur within the same hadron-hadron collision. This is referred to as DPS. Tunes that are constructed by fitting DPS-sensitive observables are referred to as “DPS tunes”. Ultimately, one universal tune that simultaneously accurately describes observables in hard scattering events, as well as MB collisions, is desirable.

The goals of this paper are to produce improved 13$\,\text {TeV}$ pythia8 tunes with well-motivated parameters, and to provide an investigation of the possible choices that can be made in pythia8 which simultaneously describe a wide range of UE and MB measurements and are suitable for merged configurations, where a ME calculation is interfaced to the simulation of UE contributions.

## Comparisons of predictions for UE observables from previous tunes to measurements at 13$\,\text {TeV}$

**Fig. 2:**
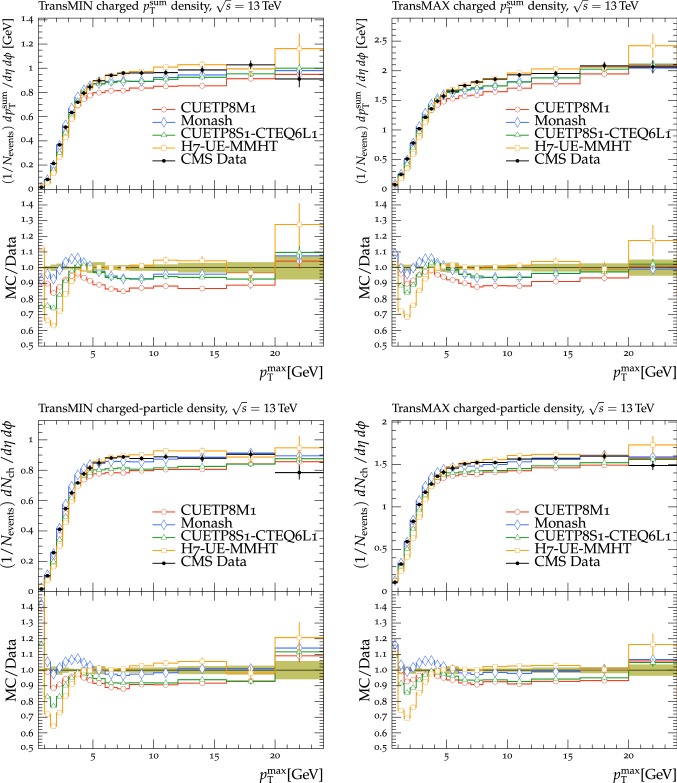
The (left column) transMIN and (right column) transMAX  charged-particle $p_{\mathrm {T}} ^\text {sum}$ (upper row), and multiplicity (lower row) densities for particles with $p_{\mathrm {T}} >0.5\,\text {GeV}$ in $|\eta | < 2.0$, as a function of the transverse momentum of the leading charged particle ($p_{\mathrm {T}} ^\text {max}$), from the CMS $\sqrt{s}=13\,\text {TeV}$ analysis [16]. The data are compared with the pythia8 tune Monash, the CMS pythia8 tunes CUETP8S1-CTEQ6L1 and CUETP8M1, and the herwig7 (labelled as “H7”) tune UE-MMHT. The ratios of the simulations to the data (MC/Data) are also shown, where the shaded band indicates the total experimental uncertainty in the data. Vertical lines drawn on the data points refer to the total uncertainty in the data. Vertical lines drawn on the MC points refer to the statistical uncertainty in the predictions. Horizontal bars indicate the associated bin width

In this section, comparisons are presented between data collected at $\sqrt{s}=13\,\text {TeV}$ and predictions from tunes obtained using fits to measurements performed at lower center-of-mass energies. Figure 2 displays comparisons of CMS data at 13$\,\text {TeV}$ [16] for the transMIN and transMAX charged-particle $p_{\mathrm {T}} ^\text {sum}$ densities, as functions of the leading charged-particle $p_{\mathrm {T}} ^\text {max}$. The data are compared with predictions from the pythia8 tunes CUETP8S1-CTEQ6L1 [5], CUETP8M1 [5], and Monash [6].

The CMS Monash-based tune CUETP8M1 does not describe the central values of the data at $\sqrt{s}=13\,\text {TeV}$ well, nor does the original Monash tune. For example, CUETP8M1 and Monash tunes do not predict enough UE activity in the region with $p_{\mathrm {T}} ^\text {max}> 5\,\text {GeV}$ (the “plateau” region) of transMIN at 13$\,\text {TeV}$, with a disagreement of $\approx$10% and $\approx$5%, respectively. The transMIN observables are very sensitive to MPI, which suggests that tune CUETP8M1 does not produce enough charged particles at 13$\,\text {TeV}$. In addition, CUETP8M1 does not provide a good fit to the jet multiplicity in $\mathrm{t}\overline{\mathrm{t}}$ production either at 8$\,\text {TeV}$ or at 13$\,\text {TeV}$ [25, 26]. High jet multiplicity $\mathrm{t}\overline{\mathrm{t}}$ events are sensitive to the modeling of the ISR. Hence, CUETP8M1 may not have the proper mixture of MPI and ISR. The ATLAS collaboration has also observed some discrepancies between the predictions of the A14 tune [12], used as standard tune for analyses of 7 and 8$\,\text {TeV}$ data, and the data at 13$\,\text {TeV}$ [27].

The CMS UE tunes were constructed by fitting CDF UE data at $\sqrt{s}=900\,\text {GeV}$ and 1.96$\,\text {TeV}$, together with CMS UE data at $\sqrt{s}=7\,\text {TeV}$. In Fig. 2 the CMS UE tunes provide a fairly good description of the 13$\,\text {TeV}$ UE data. Because the CMS UE tunes were obtained by fitting UE observables at various collision energies ($\sqrt{s}=900$, 1960, and 7000$\,\text {GeV}$), they underestimate the data at $\sqrt{s}=13\,\text {TeV}$. This might be an indication of the need to improve the energy extrapolation function implemented in pythia8 [28]. Predictions obtained with the Monash tune, which is the default pythia8 tune, slightly better reproduce the 13$\,\text {TeV}$ UE data, but is somewhat worse at describing the UE observables at $\sqrt{s}=900$ and 1960$\,\text {GeV}$ than the CMS UE tunes.

Predictions from the herwig7.1 tune UE-MMHT [3] are also shown. The H7-UE-MMHT tune was obtained by fitting UE data at $\sqrt{s}=0.9$ and 7$\,\text {TeV}$. This tune is based on the MMHT2014 PDF set [29] and is able to describe the plateau region of the UE observables at $\sqrt{s}=13\,\text {TeV}$. The part of the spectrum at $p_{\mathrm {T}} ^\text {max}> 5\,\text {GeV}$ is not well reproduced in the range of the leading charged-particle $p_{\mathrm {T}} ^\text {max}$ between 2 and 7$\,\text {GeV}$, with differences of up to 30% with respect to the data. The predictions from herwig7 achieve an overall good agreement with measurements at $\sqrt{s}=7\,\text {TeV}$ [30], while the disagreement observed for measurements at $\sqrt{s}=13\,\text {TeV}$ might indicate the need for further tuning of the new soft MPI model [30]. Since many parameters related to PS changed between herwig++ and herwig7, the CMS tunes extracted for herwig++ with the CTEQ6L1 PDF set and documented in Ref. [5] are not updated and should not be used with herwig7.

Since no currently available tune is able to optimally reproduce the UE data at $\sqrt{s}=13\,\text {TeV}$, we aim to produce improved pythia8 UE tunes.

## PDF and strong coupling values for the tunes

Two of the basic input parameters to the predictions are the choice of the order of the PDF sets and values of the strong coupling $\alpha _S$. These appear in the hard partonic MEs, the PS model, and the MPI model. The $\alpha _S$ values used in simulations at LO or NLO are typically different. Traditionally, the perturbative order of the PDF is matched to the order of the ME calculation. Merged calculations capture some higher-order corrections with respect to the formal order of the ME calculation. Merging schemes, such as the $k_{\mathrm {T}}$–MLM [31] or CKKW [32, 33], allow the combination of predictions of jet production using ME calculations with those from PS emissions for soft and collinear parton radiation at leading-log accuracy without double counting or dead regions. Merging can be applied also for processes generated at NLO. Using the same PDF set and $\alpha _S$ value in the ME calculations and in the simulation of the various components of the PS is advocated in Ref. [34], and by the herwig7 and sherpa Collaborations, especially when the PS simulation is merged with calculations of higher-order MEs. The PDF used for the hard process is constrained by the accuracy of the ME calculation. If we require the PDF to match between the ME and PS, simulations with a (N)NLO ME will also require a (N)NLO PDF in the PS. Depending on the process, this may not have a significant effect. For PS MC event generators, different strategies are adopted; CMS [5] and ATLAS [12] tunes are traditionally based on LO PDFs, pythia8 [6] tunes are mostly based on LO PDFs, new sherpa [4] tunes are based on NNLO PDFs, and herwig7 [30] provide tunes based on NLO PDFs. The usage of a LO PDF set in the UE simulation is motivated by the fact that MPI processes occur at very low energy scales, where a physical (positive) gluon distribution is required by the parton shower. However, there is no consensus on the choice of the order of the PDF. For example, in the NNPDF3.1 set at NNLO, the gluon distribution remains physical even at very low scales.

In the pythia8 tunes produced prior to this paper, the values used for $\alpha _S$ were often not the same as those used in the PDFs. For example, in the Monash tune, the FSR $\alpha _S (m_{\mathrm{Z}{}{}})$, set to 0.1365, is obtained by fitting pythia8 predictions to LEP event-shape measurements [6], the ISR $\alpha _S (m_{\mathrm{Z}{}{}})$ is assumed to be equal to FSR $\alpha _S (m_{\mathrm{Z}{}{}})$, and the hard scattering and MPI $\alpha _S (m_{\mathrm{Z}{}{}})$ is set to 0.13 according to the value used in the LO PDF set. Even though the $\alpha _S$ values are free parameters in event generators and various possibilities are viable, the usual course is to choose them consistent with the value used by the PDF set.

In this paper, a collection of new tunes is presented for PDF sets that are evaluated at different accuracies and tested against observables of MB, UE, and hard processes. The NNPDF3.1 PDF sets at the LO, NLO, and NNLO accuracy are used [17]. The LO PDF set uses an $\alpha _S (m_{\mathrm{Z}{}{}})$ value of 0.13, while 0.118 [35] is the $\alpha _S (m_{\mathrm{Z}{}{}})$ value used for the NLO and NNLO PDF sets. None of the central values of the PDF sets have negative values for any parton flavor in the phase space relevant for comparisons. Special care is required when applying these tunes at high-x regions, where the parton distributions in NNPDF3.1 NLO and NNLO PDF may become negative, which implies an unphysical (negative) value of the calculated cross sections.

The UE simulation is performed by pythia8, together with PS merged with a calculation of a higher-order or a multileg ME provided by external programs, such as powheg [36] or madgraph5_amc@NLO (mg5_amc) [37]. The issue of combining external ME calculations with PS contributions is addressed by the merging procedure. The procedures considered in this paper are the “FxFx” [38] or the “POWHEG” [39] methods for merging higher-order (NLO) MEs to PS and the “MLM” method [31].

During this study, we also investigated the effect of imposing an additional rapidity (*y*) ordering to ISR in these merging calculations. The pythia8 Monash tune includes a rapidity ordering for both ISR and MPI. The rapidity ordering acts as an extra constraint on the $p_{\mathrm {T}}$-ordered emissions, thus reducing the phase space for parton emission.

## New CMS pythia8 tunes at 13$\,\text {TeV}$

In the following, a set of new 13$\,\text {TeV}$ pythia8.226 tunes is presented with different choices of values of the strong coupling used in the modeling of the ISR, FSR, hard scattering, and MPI, as well as the order of its evolution as a function of the four-momentum squared $Q^2$. We distinguish the new tunes according to the order of the PDF set used: LO-PDF, NLO-PDF, or NNLO-PDF. The tunes are labeled as CPi, where CP stands for “CMS pythia8 ” and i is a progressive number from 1 to 5. Only five parameters related to the simulation of MPI, to the overlap matter distribution function [40], and to the amount of CR are constrained for the new CMS tunes. In all tunes, we use the MPI-based CR model [41]. The CP tunes are multipurpose tunes, aiming for a consistent description of UE and MB observables at several collision energies and a reliable prediction of the UE simulation in various processes when merged with higher-order ME calculations.

The settings, used in the determination of the new CMS pythia8 UE tunes, are as follows:

- Tune CP1 uses the NNPDF3.1 PDF set at LO, with $\alpha _S$ values used for the simulation of MPI, hard scattering, FSR, and ISR equal to, respectively, 0.13, 0.13, 0.1365, and 0.1365, and running according to an LO evolution.
- Tune CP2 is a slight variation with respect to CP1, uses the NNPDF3.1 PDF set at LO, with $\alpha _S$ values used for the simulation of MPI, hard scattering, FSR, and ISR contributions equal to 0.13, and running according to an LO evolution.
- Tune CP3 uses the NNPDF3.1 PDF set at NLO, with $\alpha _S$ values used for the simulation of MPI, hard scattering, FSR, and ISR contributions equal to 0.118, and running according to an NLO evolution.
- Tune CP4 uses the NNPDF3.1 PDF set at NNLO, with $\alpha _S$ values used for the simulation of MPI, hard scattering, FSR, and ISR contributions equal to 0.118, and running according to an NLO evolution.
- Tune CP5 has the same settings as CP4, but with the ISR emissions ordered according to rapidity.

The parameters related to the simulation of the hadronization and beam remnants are not varied in the fits and are kept fixed to the values of the Monash tune. The overlap distribution between the two colliding protons is modeled according to a double-Gaussian functional form with the parameters coreRadius and coreFraction. This parametrization of the transverse partonic overlap of two protons identifies an inner, denser part, the so-called core, and an outer less dense part. The coreRadius parameter represents the width of the core and the coreFraction, the fraction of quark and gluon content enclosed in the core. A double-Gaussian function is preferred for modeling the proton overlap over the negative exponential used in some previous tunes. Tunes using a double-Gaussian function tend to better reproduce the cross sections measured by the CMS experiment at $\sqrt{s}=7\,\text {TeV}$ [10], simultaneously as a function of charged-particle multiplicity and transverse momenta.

The parameter that determines the amount of simulated CR in the MPI-based model is varied in the fits. A small (large) value of the final-state CR parameter tends to increase (reduce) the final particle multiplicities.

The new CMS pythia8 tunes are extracted by varying the parameters listed in Table 1 and by fitting UE observables at various collision energies. In the fitting procedure, we use the charged-particle and $p_{\mathrm {T}} ^\text {sum}$ densities, measured in transMIN and transMAX regions as a function of $p_{\mathrm {T}} ^\text {max}$, as well as the charged-particle multiplicity as a function of pseudorapidity $\eta$, measured by CMS at $\sqrt{s}=13\,\text {TeV}$ [16, 18]. In addition, we also use the charged-particle and $p_{\mathrm {T}} ^\text {sum}$ densities as a function of the leading charged-particle $p_{\mathrm {T}}$, measured in transMIN and transMAX by CMS at $\sqrt{s}=7\,\text {TeV}$ [10] and by CDF at $\sqrt{s}=1.96\,\text {TeV}$ [9].

**Table 1:** Parameters in the pythia8 MC event generator together with the PDFs determine the energy dependence of MPI, the overlap matter distribution function, and the amount of simulated color reconnection. The parameter ranges used for the fits are also listed

Parameter description	Name in pythia8	Range considered
MPI threshold ($\text {GeV}$ ), pT0Ref, at $\sqrt{s}=\sqrt{s_0}$	MultipartonInteractions:pT0Ref	1.0–3.0
Exponent of $\sqrt{s}$ dependence, $\epsilon$	MultipartonInteractions:ecmPow	0.0–0.3
Matter fraction contained in the core	MultipartonInteractions:coreFraction	0.1–0.95
Radius of the core	MultipartonInteractions:coreRadius	0.1–0.8
Range of color reconnection probability	ColorReconnection:range	1.0–9.0

Tunes are determined by generating sets of predictions using the rivet[42] (version 2.5.2) and the professor[43] (version 1.4.0) frameworks with around 150 different choices of the five parameter values used in the event simulation. The predictions form a grid in the five-dimensional parameter space which is fitted using a third-order polynomial function. The uncertainty introduced in the fitted parameters due to the interpolation procedure is negligible compared with the quoted tune uncertainty. Results are found to be stable if one decreases this number to 100 or increases to 200, or uses a fourth-order polynomial function for the grid interpolation. The generated inelastic events include ND and diffractive (DD$+$SD$+$CD) contributions. The UE observables used to determine the tunes are sensitive to diffractive contributions only at very small $p_{\mathrm {T}} ^\text {max}$ values (<3$\,\text {GeV}$). The ND component is dominant for $p_{\mathrm {T}} ^\text {max}$ values greater than $\approx$3.0$\,\text {GeV}$, since the cross section of the diffractive components rapidly decreases as a function of the exchanged $p_{\mathrm {T}}$. Minimum-bias observables, such as the inclusive charged-particle multiplicity as a function of $\eta$, are sensitive to all contributions over the whole spectrum.

The fit is performed by minimizing the $\chi ^2$ function

1 $$\begin{aligned} \chi ^2(p)=\sum _{\text {O}_j} \sum _{i}\frac{(f_{i,\text {O}_j}(p)-R_{i,\text {O}_j})^2}{\varDelta _{i,\text {O}_j}^2} \end{aligned}$$

where the sum runs over each bin *i* of every observable O$_\mathrm {j}$. The $f_\mathrm {i}(p)$ functions represent a parametrization of the dependence of the predictions in bin *i* on the tuning parameters, $R_\mathrm {i}$ is the value of the measured observable in bin *i*, and $\varDelta _\mathrm {i}$ is the total experimental uncertainty of $R_\mathrm {i}$. The best fit values of the tuned parameters are shown in Table 2 for CP1 and CP2, i.e., the tunes using LO PDF sets, and in Table 3 for CP3, CP4, CP5, i.e., the tunes using NLO or NNLO PDF sets. Uncertainties in the parameters of these tunes are discussed in Appendix A. No correlation across bins is included in the minimized $\chi ^2$ function.

**Fig. 3:**
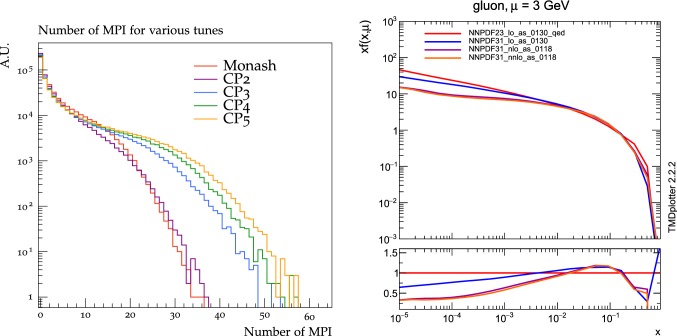
Distribution of number of MPI simulated by the tunes Monash, CP2, CP3, CP4, and CP5 (left). Gluon distribution function at a reference scale of $\mu = 3\,\text {GeV}$ (right) for the NNPDF2.3LO PDF set and the different versions of the NNPDF3.1 PDF set: LO, NLO, and NNLO. The ratio of NNPDF3.1 gluon distribution functions to the NNPDF2.3LO gluon distribution function are also shown

The value of pT0Ref and its energy dependence is very different between tunes based on LO PDF sets and tunes based on NLO or NNLO PDFs. While pT0Ref is around 2.3–2.4$\,\text {GeV}$ for CP1 and CP2 tunes with $\epsilon \approx 0.14$–0.15, CP3, CP4, and CP5 tunes prefer much lower values for both pT0Ref ($\approx$1.4–1.5) and $\epsilon$ ($\approx$0.03–0.04). A value of $\epsilon$ of $\approx$0.03–0.04 corresponds to a very weak energy dependence of the threshold of the MPI cross section. These results can be understood by considering the shapes of the gluon densities at small *x* for the different PDF sets. In order to describe the UE observables, the rapidly increasing gluon densities at small *x* in LO PDF sets favor large values of pT0Ref. Meanwhile NLO and NNLO PDF sets, whose gluon densities are more flat at low *x*, need higher contributions of MPI, i.e., a small value of pT0Ref. Figure 3 shows the number of MPI observed for the various tunes and the gluon distribution at a reference scale of $\mu = 3\,\text {GeV}$ for various NNPDF versions. The larger number of simulated MPI for NLO and NNLO tunes with respect to LO tunes is apparent.

We have found that the values of pT0Ref and $\epsilon$ also depend on the order of the running used for $\alpha _S$. In particular, fits based on NLO or NNLO PDF sets, i.e., CP3, CP4, or CP5, with an LO $\alpha _S$ running prefer even smaller values for both pT0Ref and $\epsilon$ than the ones in the tunes obtained with an NLO $\alpha _S$ running. This is because $\alpha _S$ runs faster at NLO than at LO. When $\alpha _S$ is run from the same value at the same scale ($m_{\mathrm{Z}{}{}}$), the effective coupling at low scales is larger for NLO running than for LO running. Therefore, a lower pT0Ref is needed for NLO $\alpha _S$ running than for LO $\alpha _S$ running to obtain a similar number of MPI.

For tunes based on NLO and NNLO PDF sets, the value of pT0Ref is as low as the initial scale of the PDF $Q_\text {min}^2$. For interactions occurring at $Q^2$ which are lower than $Q_\text {min}^2$, the value of the PDF is left frozen to the value assumed at the initial scale.

**Table 2:** CMS pythia8 LO-PDF tunes CP1 and CP2. Both the values at $Q = m_{\mathrm{Z}{}{}}$ and the order of running with $Q^2$ of the strong coupling $\alpha _S (m_{\mathrm{Z}{}{}})$ are listed. In these tunes, we use the Schuler-Sjöstrand diffraction model [44] and also include the simulation of CD processes. The number of degrees of freedom for tunes CP1 and CP2 is 63

pythia8 parameter	CP1	CP2
PDF Set	NNPDF3.1 LO	NNPDF3.1 LO
$\alpha _S (m_{\mathrm{Z}{}{}})$	0.130	0.130
SpaceShower:rapidityOrder	Off	Off
MultipartonInteractions:EcmRef ($\text {GeV}$ )	7000	7000
$\alpha _S ^\mathrm {ISR}(m_{\mathrm{Z}{}{}})$ value/order	0.1365/LO	0.130/LO
$\alpha _S ^\mathrm {FSR}(m_{\mathrm{Z}{}{}})$ value/order	0.1365/LO	0.130/LO
$\alpha _S ^\mathrm {MPI}(m_{\mathrm{Z}{}{}})$ value/order	0.130/LO	0.130/LO
$\alpha _S ^\mathrm {ME}(m_{\mathrm{Z}{}{}})$ value/order	0.130/LO	0.130/LO
MultipartonInteractions:pT0Ref ($\text {GeV}$ )	2.4	2.3
MultipartonInteractions:ecmPow	0.15	0.14
MultipartonInteractions:coreRadius	0.54	0.38
MultipartonInteractions:coreFraction	0.68	0.33
ColorReconnection:range	2.63	2.32
$\chi ^2$/dof	0.89	0.54

**Table 3:** CMS pythia8 NLO-PDF tune CP3 and NNLO-PDF tunes CP4 and CP5. Both the values at $Q = m_{\mathrm{Z}{}{}}$ and the order of running with $Q^2$ of the strong coupling $\alpha _S$ are listed. In these tunes, we use the Schuler-Sjöstrand diffraction model [44] and also include the simulation of CD processes. The number of degrees of freedom for tunes CP3, CP4, and CP5 is 63

pythia8 parameter	CP3	CP4	CP5
PDF set	NNPDF3.1 NLO	NNPDF3.1 NNLO	NNPDF3.1 NNLO
$\alpha _S (m_{\mathrm{Z}{}{}})$	0.118	0.118	0.118
SpaceShower:rapidityOrder	off	off	on
MultipartonInteractions:EcmRef ($\text {GeV}$ )	7000	7000	7000
$\alpha _S ^\mathrm {ISR}(m_{\mathrm{Z}{}{}})$ value/order	0.118/NLO	0.118/NLO	0.118/NLO
$\alpha _S ^\mathrm {FSR}(m_{\mathrm{Z}{}{}})$ value/order	0.118/NLO	0.118/NLO	0.118/NLO
$\alpha _S ^\mathrm {MPI}(m_{\mathrm{Z}{}{}})$ value/order	0.118/NLO	0.118/NLO	0.118/NLO
$\alpha _S ^\mathrm {ME}(m_{\mathrm{Z}{}{}})$ value/order	0.118/NLO	0.118/NLO	0.118/NLO
MultipartonInteractions:pT0Ref ($\text {GeV}$ )	1.52	1.48	1.41
MultipartonInteractions:ecmPow	0.02	0.02	0.03
MultipartonInteractions:coreRadius	0.54	0.60	0.76
MultipartonInteractions:coreFraction	0.39	0.30	0.63
ColorReconnection:range	4.73	5.61	5.18
$\chi ^2$/dof	0.76	0.80	1.04

**Fig. 4:**
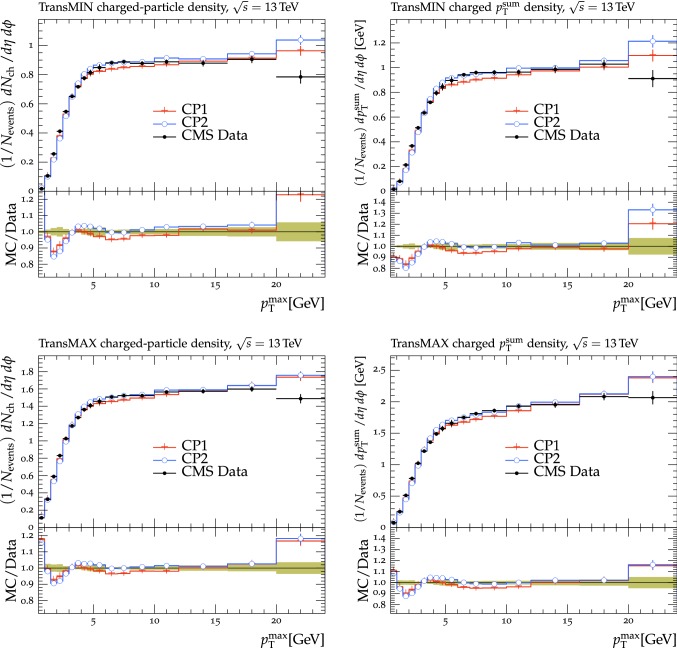
The transMIN (upper left) charged-particle and charged $p_{\mathrm {T}} ^\text {sum}$ (upper right) densities and the transMAX (lower left) charged-particle and charged $p_{\mathrm {T}} ^\text {sum}$ (lower right) densities, as a function of the transverse momentum of the leading charged particle, $p_{\mathrm {T}} ^\text {max}$, from the CMS $\sqrt{s}=13\,\text {TeV}$ analysis [16]. Charged hadrons are measured with $p_{\mathrm {T}} >0.5\,\text {GeV}$ in $|\eta |< 2.0$. The transMIN densities are more sensitive to the MPI, whereas the transMAX densities are more sensitive to ISR and FSR. The data are compared with the CMS pythia8 LO-PDF tunes CP1 and CP2. The ratios of the simulations to the data (MC/Data) are also shown, where the shaded band indicates the total experimental uncertainty in the data. Vertical lines drawn on the data points refer to the total uncertainty in the data. Vertical lines drawn on the MC points refer to the statistical uncertainty in the predictions. Horizontal bars indicate the associated bin width

**Fig. 5:**
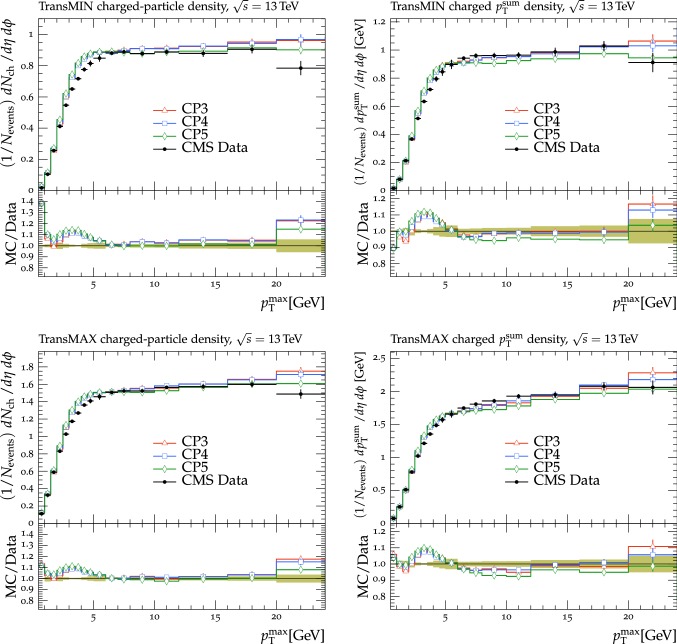
The transMIN (upper left) charged-particle and charged $p_{\mathrm {T}} ^\text {sum}$ (upper right) densities and the transMAX (lower left) charged-particle and charged $p_{\mathrm {T}} ^\text {sum}$ (lower right) densities, as a function of the transverse momentum of the leading charged particle, $p_{\mathrm {T}} ^\text {max}$, from the CMS $\sqrt{s}=13\,\text {TeV}$ analysis [16]. Charged hadrons are measured with $p_{\mathrm {T}} >0.5\,\text {GeV}$ in $|\eta |< 2.0$. The data are compared with the CMS pythia8 (N)NLO-PDF tunes CP3, CP4, and CP5. The ratios of simulations to the data (MC/Data) are also shown, where the shaded band indicates the total experimental uncertainty in the data. Vertical lines drawn on the data points refer to the total uncertainty in the data. Vertical lines drawn on the MC points refer to the statistical uncertainty in the predictions. Horizontal bars indicate the associated bin width

**Fig. 6:**
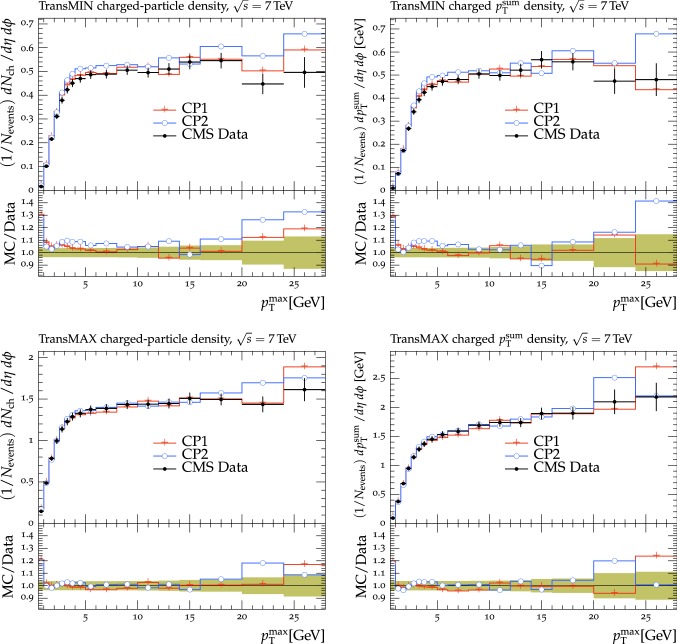
The transMIN (upper left) charged-particle and charged $p_{\mathrm {T}} ^\text {sum}$ (upper right) densities and the transMAX (lower left) charged-particle and charged $p_{\mathrm {T}} ^\text {sum}$ (lower right) densities, as a function of the transverse momentum of the leading charged particle, $p_{\mathrm {T}} ^\text {max}$, from the CMS $\sqrt{s}=7\,\text {TeV}$ analysis [10]. Charged hadrons are measured with $p_{\mathrm {T}} >0.5\,\text {GeV}$ in $|\eta |< 0.8$. The data are compared with the CMS pythia8 LO-PDF tunes CP1 and CP2. The ratios of simulations to the data (MC/Data) are also shown, where the shaded band indicates the total experimental uncertainty in the data. Vertical lines drawn on the data points refer to the total uncertainty in the data. Vertical lines drawn on the MC points refer to the statistical uncertainty in the predictions. Horizontal bars indicate the associated bin width

**Fig. 7:**
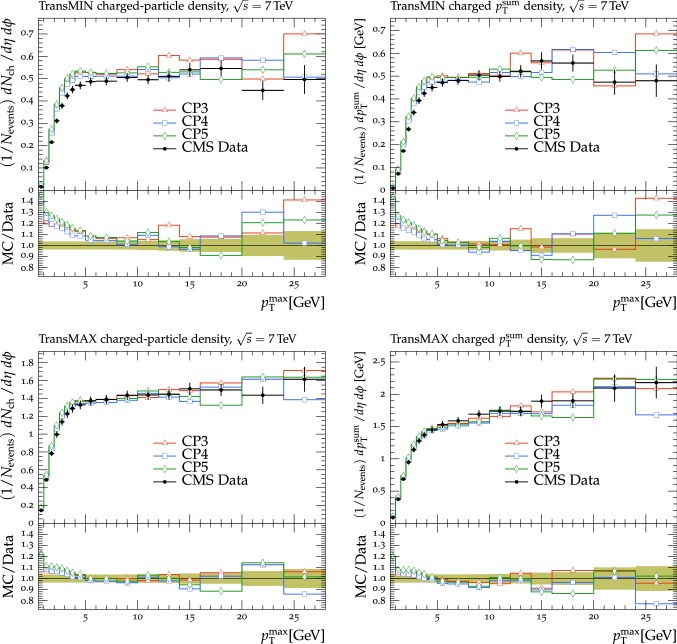
The transMIN (upper left) charged-particle and charged $p_{\mathrm {T}} ^\text {sum}$ (upper right) densities and the transMAX (lower left) charged-particle and charged $p_{\mathrm {T}} ^\text {sum}$ (lower right) densities, as a function of the transverse momentum of the leading charged particle, $p_{\mathrm {T}} ^\text {max}$, from the CMS $\sqrt{s}=7\,\text {TeV}$ analysis [10]. Charged hadrons are measured with $p_{\mathrm {T}} >0.5\,\text {GeV}$ in $|\eta |< 0.8$. The data are compared with the CMS pythia8 (N)NLO-PDF tunes CP3, CP4, and CP5. The ratios of simulations to the data (MC/Data) are also shown, where the shaded band indicates the total experimental uncertainty in the data. Vertical lines drawn on the data points refer to the total uncertainty in the data. Vertical lines drawn on the MC points refer to the statistical uncertainty in the predictions. Horizontal bars indicate the associated bin width

**Fig. 8:**
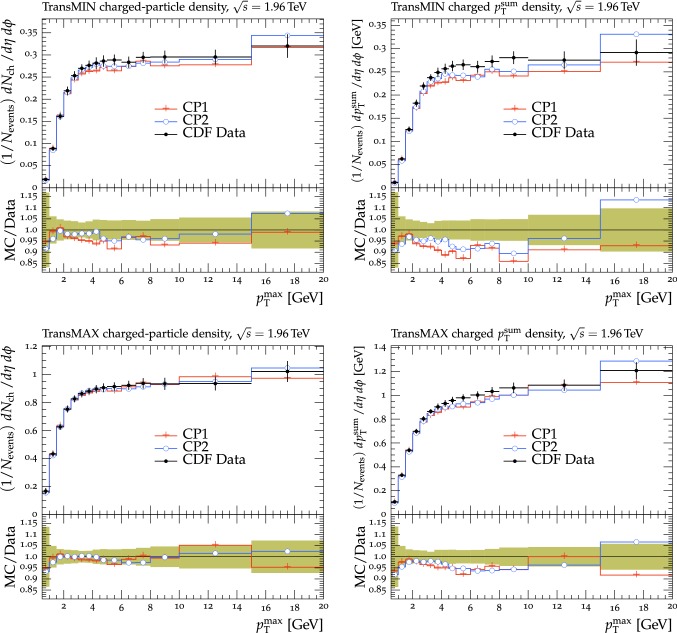
The transMIN (upper left) charged-particle and charged $p_{\mathrm {T}} ^\text {sum}$ (upper right) densities and the transMAX (lower left) charged-particle and charged $p_{\mathrm {T}} ^\text {sum}$ (lower right) densities, as a function of the transverse momentum of the leading charged particle, $p_{\mathrm {T}} ^\text {max}$, from the CDF $\sqrt{s}=1.96\,\text {TeV}$ analysis [9]. Charged hadrons are measured with $p_{\mathrm {T}} >0.5\,\text {GeV}$ in $|\eta |< 0.8$. The data are compared with the CMS pythia8 LO-PDF tunes CP1 and CP2. The ratios of simulations to the data (MC/Data) are also shown, where the shaded band indicates the total experimental uncertainty in the data. Vertical lines drawn on the data points refer to the total uncertainty in the data. Vertical lines drawn on the MC points refer to the statistical uncertainty in the predictions. Horizontal bars indicate the associated bin width

**Fig. 9:**
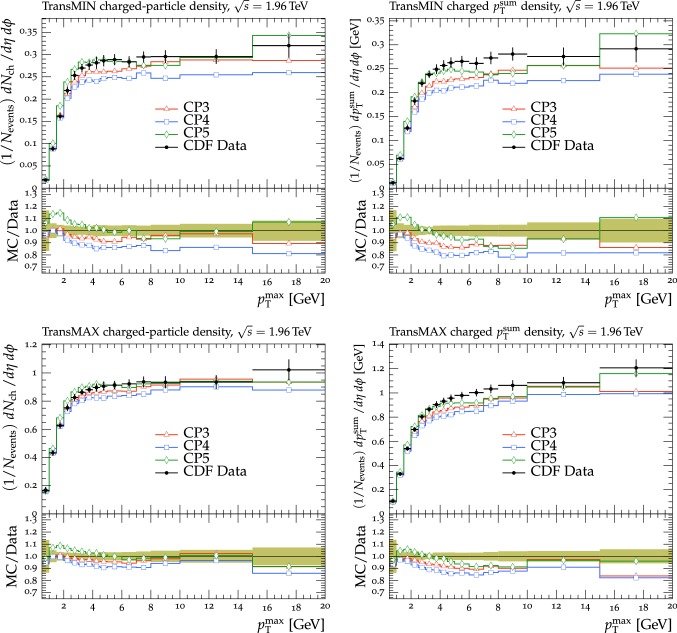
The transMIN (upper left) charged-particle and charged $p_{\mathrm {T}} ^\text {sum}$ (upper right) densities and the transMAX (lower left) charged-particle and charged $p_{\mathrm {T}} ^\text {sum}$ (lower right) densities, as a function of the transverse momentum of the leading charged particle, $p_{\mathrm {T}} ^\text {max}$, from the CDF $\sqrt{s}=1.96\,\text {TeV}$ analysis [9]. Charged hadrons are measured with $p_{\mathrm {T}} >0.5\,\text {GeV}$ in $|\eta |< 0.8$. The data are compared with the CMS pythia8 (N)NLO-PDF tunes CP3, CP4, and CP5. The ratios of simulations to the data (MC/Data) are also shown, where the shaded band indicates the total experimental uncertainty in the data. Vertical lines drawn on the data points refer to the total uncertainty in the data. Vertical lines drawn on the MC points refer to the statistical uncertainty in the predictions. Horizontal bars indicate the associated bin width

**Fig. 10:**
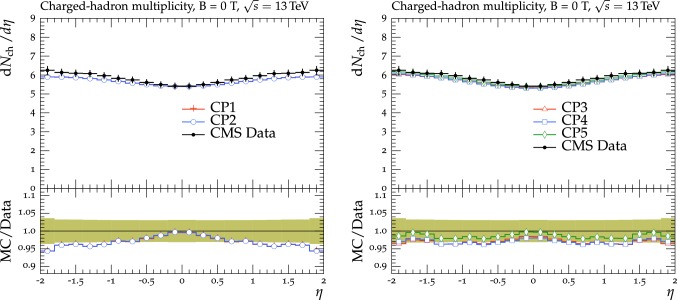
The pseudorapidity distribution of charged hadrons measured in $|\eta | < 2$ for an inclusive selection in inelastic proton-proton collisions, with zero magnetic field strength (B = 0 T), from the CMS $\sqrt{s}=13\,\text {TeV}$ analysis [18]. The data are compared with the CMS pythia8 LO-PDF tunes CP1 and CP2 (left), and with the CMS pythia8 NLO-PDF tune CP3 and the CMS pythia8 NNLO-PDF tunes CP4 and CP5 (right). The ratios of simulations to the data (MC/Data) are also shown, where the shaded band indicates the total experimental uncertainty in the data. Vertical lines drawn on the data points refer to the total uncertainty in the data. Vertical lines drawn on the MC points refer to the statistical uncertainty in the predictions. Horizontal bars indicate the associated bin width

The contribution from CR also changes among the different tunes and depends on the choice of PDF and its order. In particular, the amount of CR is also affected by the shape of the PDFs at small fractional momenta *x*.

Parameters related to the overlap matter distribution function differ between the different tunes. They are strongly correlated with the other UE parameters governing the MPI and CR contributions. In general, for a given value of the matter fraction (coreFraction), MPI contributions increase for decreasing values of the core radius (coreRadius). The inclusion of the rapidity ordering for ISR in tune CP5 impacts the UE observables by reducing the number of charged particles, and needs to be compensated by a larger amount of MPI contributions.

The $\chi ^2$ per degree of freedom (dof) listed in Tables 2 and 3 refers to the quantity $\chi ^2(p)$ in Eq. (1), divided by dof in the fit. The eigentunes (Appendix A) correspond to the tunes in which the changes in the $\chi ^2$ ($\varDelta \chi ^2$) of the fit relative to the best fit value equals the $\chi ^2$ value obtained in the tune, i.e., $\varDelta \chi ^2_\text {min}=\chi ^2$. Such a variation of the $\chi ^2$ produces a tune whose uncertainty bands are roughly the same as the uncertainties in the fitted data points. This is the main motivation why this choice of variation was considered. For all tunes in Tables 2 and 3, the fit quality is good, with $\chi ^2$/dof values very close to 1.

Figures 4, 5, 6 and 7 show comparisons of the UE observables measured at various collision energies to predictions from the new tunes. Figures 4 and 5 compare the charged-particle and $p_{\mathrm {T}} ^\text {sum}$ densities measured at $\sqrt{s}=13\,\text {TeV}$ by the CMS experiment [16] in the transMIN and transMAX regions to predictions from the LO-PDF-based tunes and the higher-order-PDF-based tunes. Figures 6 and 7 compare the charged-particle and $p_{\mathrm {T}} ^\text {sum}$ densities measured at $\sqrt{s}=7\,\text {TeV}$ by the CMS experiment [10] in the transMIN and transMAX regions to predictions from the LO-PDF-based tunes and the higher-order-PDF-based tunes. In Figs. 8 and 9 similar comparisons are shown for the observables measured at $\sqrt{s}=1.96\,\text {TeV}$ by the CDF experiment [9] in the transMIN and transMAX regions. All predictions reproduce well the UE observables at $\sqrt{s}=1.96$, 7, and 13$\,\text {TeV}$. Predictions from LO tunes are slightly better than the higher-order tunes in describing the energy dependence of the considered UE measurements.

In the region of small $p_{\mathrm {T}} ^\text {max}$ values ($p_{\mathrm {T}} ^\text {max}<3\,\text {GeV}$), where contributions from diffractive processes are relevant, the predictions do not always reproduce the measurements and exhibit discrepancies up to 20%. Predictions from all of the new tunes cannot reproduce the UE data measured at $\sqrt{s} = 300$ and 900$\,\text {GeV}$ [9].

Figure 10 shows the charged-particle multiplicity as a function of pseudorapidity for charged particles in $|\eta | < 2$ measured by the CMS experiment at $\sqrt{s}=13\,\text {TeV}$ [18] in MB events. These events were recorded with no magnetic field, so all particles irrespective of their $p_{\mathrm {T}}$ are measured. Data are compared with the predictions of the new pythia8 tunes. All of them are able to reproduce the measurement at the same level of agreement, independently of the PDF used for the UE simulation. We could not find any MB or UE observable where the level of agreement between data and predictions from the different tunes is significantly different.

## Validation of the new pythia8 tunes

In this section, comparisons of the predictions obtained with the new tunes to various experimental measurements performed by the CMS experiment are provided. Unless otherwise stated, the comparisons are made at $\sqrt{s}=13\,\text {TeV}$. We compare the CMS UE tunes with MB and UE data measured at central and forward pseudorapidities that are not used in the fits. We examine how well multijet, Drell–Yan, and top quark observables are predicted by MC simulations using higher-order ME generators merged with pythia8 with the various new tunes.

### Comparisons using event-shape observables

In this subsection, predictions of the new tunes are compared to event-shape observables measured at LEP, in electron-positron collisions. These observables are particularly sensitive to the value of $\alpha _S ^\mathrm {FSR}(m_{\mathrm{Z}{}{}})$. Given the leptonic initial state, there is no effect coming from the values of the MPI, color reconnection, and ISR parameters.

When predictions with pythia  8 are used, an optimal value of $\alpha _S ^{\mathrm {FSR}}(m_{\mathrm{Z}{}{}})\sim 0.13$ is found, which best describes these observables, independent of the PDF used for the modeling of the PS evolution.

**Fig. 11:**
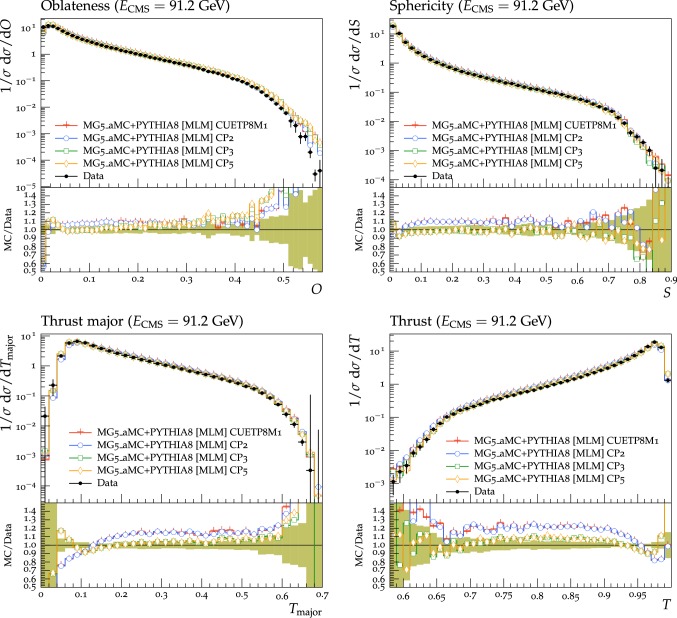
The normalized cross sections as a function of event-shape variables, oblateness (upper left), sphericity (upper right), thrust (lower left), and thrust major (lower right) from the ALEPH $\sqrt{s} =91.2\,\text {GeV}$ analysis [45], compared with the predictions by mg5_amc $+$ pythia8 with $k_{\mathrm {T}}$–MLM merging, for tunes CP2, CP3, and CP5. The ratio of the simulations to the data (MC/Data) is also shown, where the shaded band indicates the total experimental uncertainty in the data. Vertical lines drawn on the data points refer to the total uncertainty in the data. Vertical lines drawn on the MC points refer to the statistical uncertainty in the predictions. Horizontal bars indicate the associated bin width

**Fig. 12:**
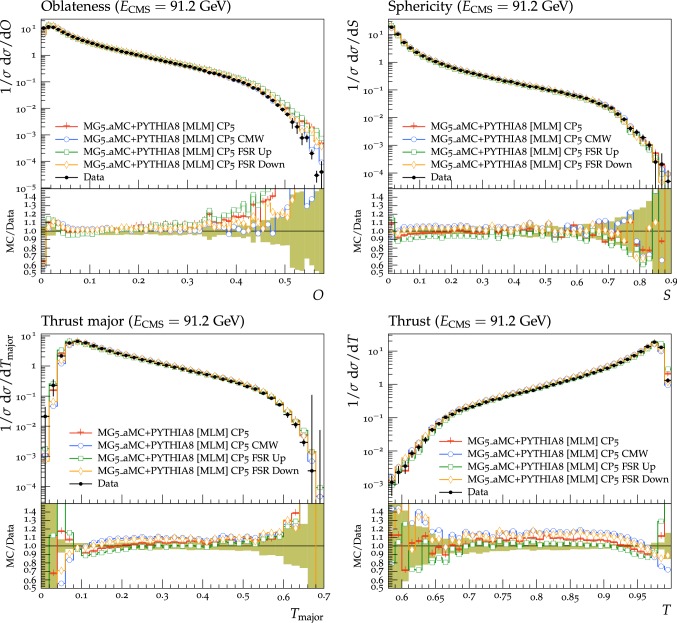
The normalized cross sections as a function of event-shape variables, oblateness (upper left), sphericity (upper right), thrust (lower left), and thrust major (lower right) from the ALEPH $\sqrt{s} =91.2\,\text {GeV}$ analysis [45], compared with the predictions by mg5_amc $+$ pythia8 with $k_{\mathrm {T}}$–MLM merging, for tune CP5, CP5 with CMW rescaling, CP5 FSR up, and CP5 FSR down. The ratio of the simulations to the data (MC/Data) is also shown, where the shaded band indicates the total experimental uncertainty in the data. Vertical lines drawn on the data points refer to the total uncertainty in the data. Vertical lines drawn on the MC points refer to the statistical uncertainty in the predictions. Horizontal bars indicate the associated bin width

Figures 11 and 12 display the oblateness (*O*), sphericity (*S*), thrust (*T*), and thrust major ($T_\text {major}$), measured in ${\mathrm{{\mathrm{e}{}{}}}^{+}} {\mathrm{{\mathrm{e}{}{}}}^{-}} \rightarrow {\mathrm{Z}{}{}} \upgamma ^{{*}} \rightarrow {\mathrm{q}{}{}} {\overline{\mathrm{{\mathrm{q}{}{}}}}{}{}}$ final states at $\sqrt{s} = 91.2$ $\,\text {GeV}$ by the ALEPH experiment [45]. These observables measure the topology of the event. An isotropic event would have a value of *T* close to 0.5, while values of *T* close to 1 correspond to 2-jet events.

Predictions obtained with mg5_amc with up to 4 partons in the final state, and interfaced with the UE from the tune CUETP8M1 and the new pythia  8 tunes CP2, CP3, and CP5 are considered (Fig. 11). Predictions using the tune CP2 do not describe the event-shape observables very well, with discrepancies with the data up to 30% in the *T* and $T_\text {major}$. In particular, tune CP2 predicts too many isotropical events. A similar description is obtained for predictions of mg5_amc $+$ pythia  8 with the tune CUETP8M1. A better agreement in the event-shape variables is observed for predictions using tune CP3 and CP5. A correct description of event-shape observables strongly depends on the value of the FSR strong coupling. The observations above indicate that when merged configurations are considered, i.e., mg5_amc $+$ pythia8, where partons at higher multiplicities in the final state are simulated at the ME level, the description of event-shape observables degrades. A value of $\alpha _S ^\mathrm {FSR}(m_{\mathrm{Z}{}{}})\sim 0.13$ generally overestimates the number of final-state partons, while a lower $\alpha _S ^\mathrm {FSR}(m_{\mathrm{Z}{}{}})\sim 0.12$ performs better.

At large values of *T*, where the hadronization effects become relevant, we observe a large difference between predictions from tunes using a small $\alpha _S ^\mathrm {FSR}$ (CP3 and CP5) and tunes using a large $\alpha _S ^\mathrm {FSR}$ (CP2 and CUETP8M1). These differences may be due to the interplay between the value of the strong coupling and the hadronization. Analyses particularly sensitive to hadronization should carefully evaluate the corresponding systematic uncertainties. In some cases retuning hadronization parameters may be desired.

We also compared mg5_amc $+$ pythia  8 with CP5, and CP5 with CMW rescaling [46] (Fig. 12). Apart from *T*, for all shape variables considered, CP5 without CMW rescaling describes the data better.

### Comparisons using MB and other UE observables

**Fig. 13:**
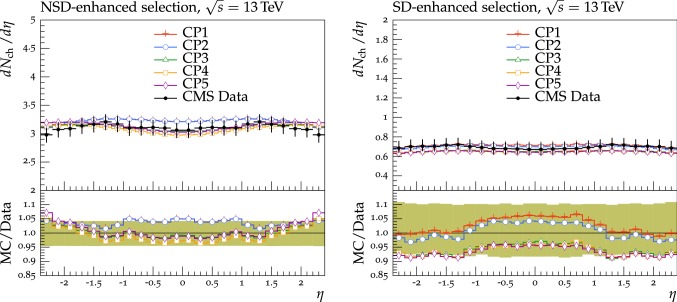
The pseudorapidity distribution ($p_{\mathrm {T}} >0.5\,\text {GeV}$, $|\eta |<2.4$) for the NSD-enhanced (left) and the SD-enhanced (right) event selection of charged particles in inelastic proton-proton collisions, from the CMS $\sqrt{s}=13\,\text {TeV}$ analysis [24]. The data are compared with the CMS pythia8 LO-PDF tunes CP1 and CP2, the CMS pythia8 NLO-PDF tune CP3, and the CMS pythia8 NNLO-PDF tunes CP4 and CP5. The ratio of the simulations to the data (MC/Data) is also shown, where the shaded band indicates the total experimental uncertainty in the data. Vertical lines drawn on the data points refer to the total uncertainty in the data. Vertical lines drawn on the MC points refer to the statistical uncertainty in the predictions. Horizontal bars indicate the associated bin width

In this subsection, predictions of the new tunes are compared to the observables measured in MB collisions that are sensitive to contributions from soft emissions and MPI. Figure 13 shows the charged-particle multiplicity as a function of pseudorapidity [24] in NSD-enhanced and SD-enhanced event samples. The details of the selections can be found in Ref. [24]. These observables are sensitive to SD, CD, and DD dissociation. It is observed that predictions from all of the tunes are similar to each other and describe well the measurements for both considered selections. This shows that the number of charged particles produced in diffractive processes and inelastic collisions is simultaneously described by the new CMS tunes. Figure 13 also demonstrates that tunes based on NNPDF3.1 PDF sets at orders higher than LO adequately describe the MB data.

**Fig. 14:**
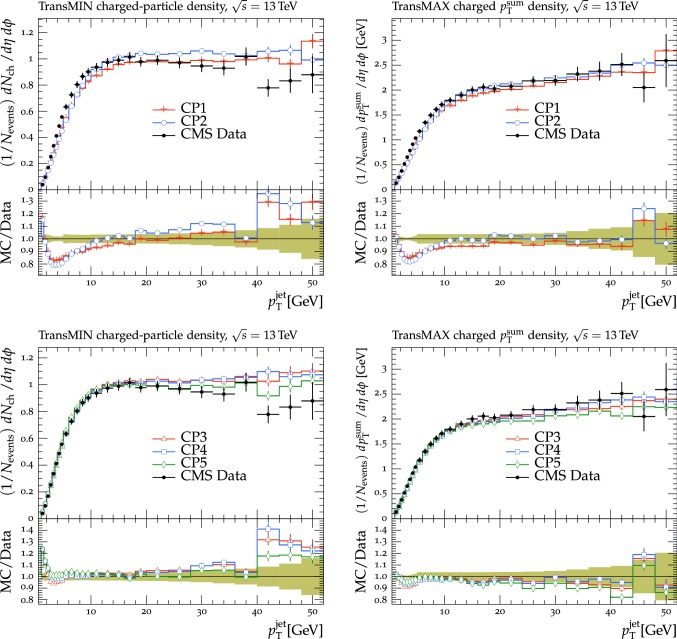
The transMIN charged-particle multiplicity (left column) and $p_{\mathrm {T}}$ sum densities (right column) for particles with $p_{\mathrm {T}} >0.5\,\text {GeV}$ in $|\eta |< 2.0$ as a function of the transverse momentum of the leading charged-particle jet, $p_{\mathrm {T}} ^\text {jet}$, from the CMS $\sqrt{s}=13\,\text {TeV}$ analysis [16]. The upper-row plots show the LO tunes, while the lower-row plots show the higher-order tunes. The ratio of the simulations to the data (MC/Data) is also shown, where the shaded band indicates the total experimental uncertainty in the data. Vertical lines drawn on the data points refer to the total uncertainty in the data. Vertical lines drawn on the MC points refer to the statistical uncertainty in the predictions. Horizontal bars indicate the associated bin width

Figure 14 shows the UE observables, i.e., charged-particle multiplicity and $p_{\mathrm {T}} ^\text {sum}$ densities [16], as a function of the $p_{\mathrm {T}}$ of the leading jet reconstructed using just charged particles. The observables shown in Fig. 14 are from events selected without requiring any NSD- or SD-enhanced selections. The CMS UE tunes describe well UE-sensitive data measured using the leading charged-particle jet for $p_{\mathrm {T}}$ $^{\text {jet}}>10\,\text {GeV}$. Tunes based on NLO or NNLO PDF sets, i.e., CP3, CP4, and CP5, describe the region at lower $p_{\mathrm {T}}$ $^{\text {jet}}$ better than CP1 and CP2, which are based on LO PDF sets. Predictions obtained with CP1 and CP2 underestimate the UE observables by about $\approx$15–20%. Predictions obtained with CP3, CP4, and CP5 describe the UE in events characterized using the leading charged particle, as well as those characterized by the leading charged-particle jet, quite well.

**Fig. 15:**
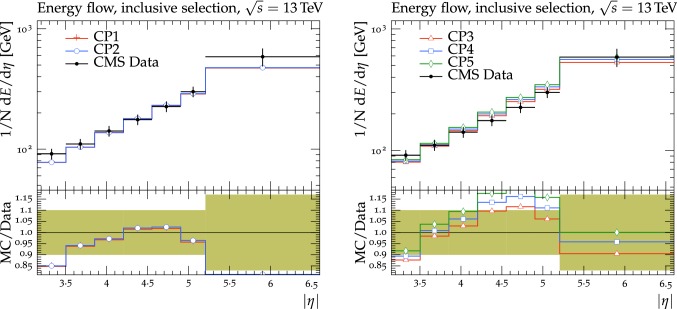
The energy flow measured in an inclusive selection as a function of pseudorapidity, from the CMS $\sqrt{s}=13\,\text {TeV}$ analysis [47]. The data are compared with the CMS pythia8 LO-PDF tunes CP1 and CP2 (left), and with the CMS pythia8 NLO-PDF tune CP3 and the CMS pythia8 NNLO-PDF tunes CP4 and CP5 (right). The ratio of the simulations to the data (MC/Data) is also shown, where the shaded band indicates the total experimental uncertainty in the data. Vertical lines drawn on the data points refer to the total uncertainty in the data. Vertical lines drawn on the MC points refer to the statistical uncertainty in the predictions. Horizontal bars indicate the associated bin width

**Fig. 16:**
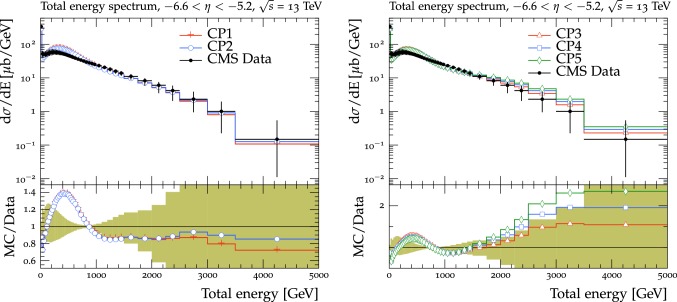
The total energy spectrum measured in the pseudorapidity interval $-6.6<\eta <-5.2$, from the CMS $\sqrt{s}=13\,\text {TeV}$ analysis [49]. The data are compared with the CMS pythia8 LO-PDF tunes CP1 and CP2 (left), and with the CMS pythia8 NLO-PDF tune CP3 and the CMS pythia8 NNLO-PDF tunes CP4 and CP5 (right). The ratio of the simulations to the data (MC/Data) is also shown, where the shaded band indicates the total experimental uncertainty in the data. Vertical lines drawn on the data points refer to the total uncertainty in the data. Vertical lines drawn on the MC points refer to the statistical uncertainty in the predictions. Horizontal bars indicate the associated bin width

Predictions for observables measured in the forward region are compared with data and shown in Figs. 15 and 16. The energy flow, defined as the average energy per event [47], as a function of $\eta$ with the Hadron Forward (HF) calorimeter [48] covering $3.15<|\eta |< 5.20$ and the CASTOR calorimeter [48] covering $-6.6<\eta <-5.2$, is well reproduced by all tunes. A different level of agreement is achieved for predictions from the new CMS tunes for the spectrum of the total energy E measured in the CASTOR calorimeter at $\sqrt{s}=13\,\text {TeV}$ [49], displayed in Fig. 16. In particular, the tunes based on LO PDF sets reproduce the energy spectrum well at large values ($\text {E}>2000\,\text {GeV}$), but have differences of up to 40% at low values ($\text {E}<800\,\text {GeV}$). The tunes using higher-order PDF sets are closer to the data at low energy values, with differences up to 20%, but tend to overestimate the energy at large values. This dissimilar behaviour is driven by the different pT0Ref values of the tunes. The fiducial inelastic cross sections [50], when two different selections are applied in the forward region, are not well reproduced by any of the new tunes or by CUETP8M1, with differences up to 10%. This might be because of the Schüler–Sjöstrand [44] diffraction model used in the simulation, which might have a suboptimal description of the low-mass diffractive components. A better description might be provided by tunes using the Donnachie–Landshoff [51] or minimum-bias Rockefeller [52] diffractive models.

### Comparisons using observables in multijet final states

In this subsection, we present comparisons of observables measured in multijet final states. For these studies, the NLO dijet MEs implemented in the powheg event generator merged with the pythia8 simulation of the PS and UE are used. The merging between the powheg ME calculations and the pythia8 UE simulation is performed using the shower-veto procedure, which rejects showers if their transverse momentum is greater than the minimal $p_{\mathrm {T}}$ of all final-state partons simulated in the ME (parameter $p_{\mathrm {T}}$ $^\text {hard}= 2\,\text {GeV}$ [53]). Variables in multijet events, such as jet transverse momenta or azimuthal dijet correlations, are expected to be less affected by MPI contributions, since jets at high $p_{\mathrm {T}}$ (>100$\,\text {GeV}$) mainly originate from the hard scattering or additional hard emissions, which are simulated in the powheg calculation by the ME formalism. However, the MPI contribution still has some impact because it adds an average energy offset to the event, which is then included in the jet reconstruction [54, 55]. The predictions reproduce well inclusive jet cross sections as a function of jet $p_{\mathrm {T}}$ at both central and forward jet rapidities, irrespective of the cone size (0.4 or 0.7) used for the jet clustering algorithm [56].

**Fig. 17:**
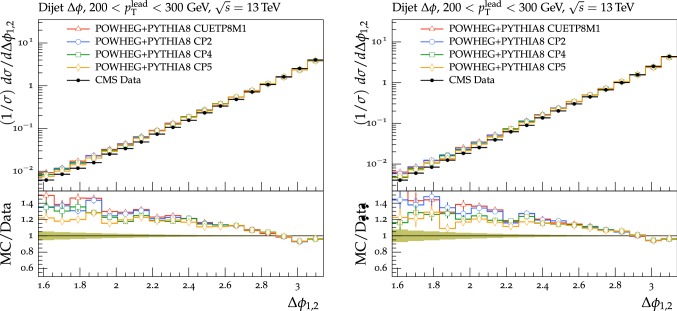
The azimuthal difference $\varDelta \phi _{1,2}$ between the leading two jets with $|\eta |<2.4$ in dijet events with leading-jet transverse momentum in the range (left) $200<p_{\mathrm {T}} ^{\text {lead}}< 300\,\text {GeV}$ and (right) $300<p_{\mathrm {T}} ^{\text {lead}}<400\,\text {GeV}$, from the CMS $\sqrt{s}=13\,\text {TeV}$ analysis [57]. The jets are reconstructed using the anti-$k_{\mathrm {T}}$ jet finding algorithm [58, 59] with a distance parameter of 0.4. The data are compared with predictions of the NLO dijet ME calculation from powheg, interfaced to the pythia8 tunes CUETP8M1, CP2, CP4, and CP5. Tunes CP1 and CP3 are not shown in the plot but present a similar behavior as tunes CP2 and CP4. The ratios of simulations to the data (MC/Data) are also shown, where the shaded band indicates the total experimental uncertainty in the data. Vertical lines drawn on the data points refer to the total uncertainty in the data. Vertical lines drawn on the MC points refer to the statistical uncertainty in the predictions. Horizontal bars indicate the associated bin width

Figure 17 shows the normalized cross section [57] as a function of the azimuthal difference $\varDelta \phi _{1,2}$ between the two leading jets for two different selections on the leading jet $p_{\mathrm {T}}$: $200<p_{\mathrm {T}} <300\,\text {GeV}$ and $300<p_{\mathrm {T}} <400\,\text {GeV}$. The results indicate that UE tunes based on an NLO evaluation of $\alpha _S (m_{\mathrm{Z}{}{}})$ describe the data better than UE tunes based on LO evolution. In particular, the better agreement is driven by the lower value of $\alpha _S ^\mathrm {ISR}(m_{\mathrm{Z}{}{}})$. In fact, predictions obtained with powheg merged with pythia8 with the CUETP8M1 or CP2 tune exhibit a strong jet decorrelation, due to a large contribution from emissions simulated from the PS, and they overestimate the cross sections at small and medium $\varDelta \phi _{1,2}$ values ($\varDelta \phi _{1,2}< 2.4$). The PS component is reduced by the lower value of $\alpha _S (m_{\mathrm{Z}{}{}})$, which increases the degree of correlation between the selected jets, resulting in a better description of the data by predictions of the CP4 and CP5 tunes. A similar outcome was also observed for an analogous measurement performed at the D0 experiment at $\sqrt{s}=1.96\,\text {TeV}$ [60]. In general, predictions obtained from powheg  $+$ pythia8 tend to differ from the data at low and intermediate $\varDelta \phi _{1,2}$ values ($\varDelta \phi _{1,2}< 2.7$) by 10–40%.

### Comparisons using observables sensitive to double-parton scattering

In this subsection, we present comparisons of predictions of the new tunes to DPS-sensitive observables measured by the CMS experiment at $\sqrt{s}=7\,\text {TeV}$ in final states with four jets (4j) [61], and with two jets originating from bottom quarks (b jets) [62] and two other jets (2b2j) [63].

The topology in the transverse plane of the physics objects measured in the final state is sensitive to contributions from DPS. In particular, the 4j analysis performed by the CMS experiment requires two jets at high $p_{\mathrm {T}}$ (hard jets) and two jets at low $p_{\mathrm {T}}$ (soft jets); the 2b2j measurement selects two jets originating from b quarks and two other jets (light-flavor jets). Both of them measured the $\varDelta$S observable, defined as:

2 $$\begin{aligned} \varDelta S=\arccos \left( \frac{\vec {p}_{\text {T,1}}\cdot \vec {p}_{\text {T,2}}}{|\vec {p}_{\text {T,1}} | |\vec {p}_{\text {T,2}} |}\right) , \end{aligned}$$

where $\vec {p}_\mathrm {T,1}$ refers to the momentum of the hard-jet or bottom jet pair system and $\vec {p}_\mathrm {T,2}$ to that of the soft-jet or light-flavor jet pair system. This variable relates the production planes of the hard (bottom) jet and soft- (light-flavor) jet pairs. Details of the event selection and of the specific analyses can be found in Refs. [61] and [63].

Assuming that the two hard scatterings occurring within the same collision are completely independent of each other, the DPS cross section for a given process can be expressed through the inclusive partonic cross sections of the two single scatterings and an effective cross section, $\sigma _{\text {eff}}$. In a geometrical approach, this cross section is related to the transverse size of the proton and to the total inelastic proton-proton (${\mathrm{p}{}{}} {\mathrm{p}{}{}}$) cross section [64, 65]. When no correlations among the partons inside the proton are present, $\sigma _{\text {eff}}$ is similar to the inelastic ${\mathrm{p}{}{}} {\mathrm{p}{}{}}$ cross section. In this simple factorized approach, one expects $\sigma _{\text {eff}}$ to be independent of the partonic final states of the two hard processes occurring within the same collision. In pythia8, the value of $\sigma _{\text {eff}}$ is calculated by dividing the ND cross section by the so-called “enhancement factor”, which depends on the parameters of the overlap matter distribution function and on pT0Ref [40]. For central ${\mathrm{p}{}{}} {\mathrm{p}{}{}}$ collisions, the enhancement factor tends to be large, translating to a lower value of $\sigma _{\text {eff}}$ and a larger DPS contribution. For peripheral interactions, enhancement factors are small, giving large values of $\sigma _{\text {eff}}$ and a small DPS contribution.

Table 4 shows the values of $\sigma _{\text {eff}}$ published by the CMS Collaboration for the 4j and the 2b2j measurements. A previous study [5] concluded that observables sensitive to semi-hard MPI and those sensitive to DPS cannot be described by a single set of parameters. Table 5 displays the $\sigma _{\text {eff}}$ values obtained from the new CMS UE tunes. The central values of $\sigma _{\text {eff}}$ are consistent among the new tunes and are slightly larger than the values of the DPS-based tunes [5].

**Fig. 18:**
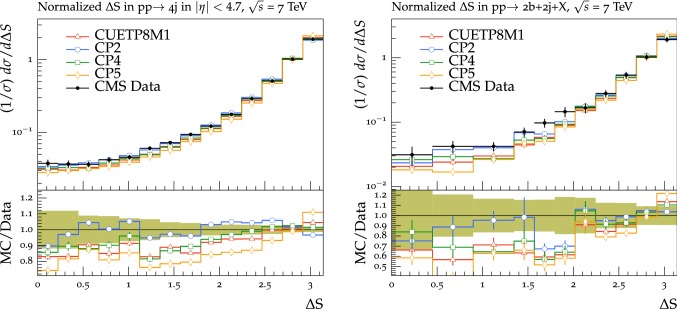
The correlation observable $\varDelta$S measured in 4j (left) and 2b2j (right) production, compared to predictions of pythia8 tunes CUETP8M1, CP2, CP4, and CP5, from the CMS $\sqrt{s}=7\,\text {TeV}$ analyses [61, 63]. Tunes CP1 and CP3 are not shown in the plot but show a similar behaviour as, respectively, tunes CP2 and CP4. The ratios of simulations to the data (MC/Data) are also shown, where the shaded band indicates the total experimental uncertainty in the data. Vertical lines drawn on the data points refer to the total uncertainty in the data. Vertical lines drawn on the MC points refer to the statistical uncertainty in the predictions. Horizontal bars indicate the associated bin width

Figure 18 shows the comparisons of predictions obtained from pythia8 with tunes CUETP8M1, CP2, CP4, and CP5 to the DPS observables measured in the 4j and 2b2j final states. Predictions from the CP2 tune based on a LO PDF set describe the central values better than the CP4 and CP5 tunes based on an NNLO PDF set or the old tune CUETP8M1. This is due to the different pT0Ref value used by CP2, CP4, and CP5, which determines the amount of simulated MPI. The value of the pT0Ref parameter is driven by the distribution of the gluon distribution function at low *x*, which is very different in LO and NNLO PDF sets. Additionally, predictions obtained with CP4 describe the DPS-sensitive observables better than CP5. This is due to the different rapidity ordering used for the PS emissions in the two tunes. By removing the rapidity ordering for the PS emissions (CP4), the simulation produces more radiation and decreases the correlation between the selected jet pairs compared to CP5. This reduced jet correlation tends to mimic a DPS event by producing low values of $\varDelta$S. We have checked that the observables sensitive to color coherence, which were measured by the CMS experiment at $\sqrt{s}=7\,\text {TeV}$ [66], are well described by predictions from both CP4 and CP5 tunes, despite the difference in the rapidity ordering of the PS simulation between the two tunes.

**Table 4:** Values of $\sigma _{\text {eff}}$ at $\sqrt{s}=7\,\text {TeV}$ published by the CMS Collaboration for the four-jet final states, obtained by fitting predictions of the pythia8 MC event generator to DPS-sensitive measured observables

Final state	Generator	$\sigma _{\text {eff}}$ (mb) ($\sqrt{s}=7\,\text {TeV}$)
4j	pythia8	19.0$^{+4.7}_{-3.0}$ [5]
2b2j	pythia8	23.2$^{+3.3}_{-2.5}$ [67]

**Table 5:** Values of $\sigma _{\text {eff}}$ at $\sqrt{s}=7$ and 13$\,\text {TeV}$ obtained with the new CMS UE tunes

	$\sqrt{s}=7\,\text {TeV}$	$\sqrt{s}=13\,\text {TeV}$
	$\sigma _{\text {eff}}$ (mb)	$\sigma _{\text {eff}}$ (mb)
CP1	26.3$^{+1.0}_{-1.7}$	27.8$^{+1.1}_{-1.4}$
CP2	24.7$^{+1.0}_{-1.6}$	26.0$^{+1.0}_{-1.3}$
CP3	24.1$^{+1.0}_{-1.5}$	25.2$^{+1.0}_{-1.3}$
CP4	23.9$^{+1.0}_{-1.5}$	25.3$^{+1.1}_{-1.4}$
CP5	24.0$^{+1.0}_{-1.6}$	25.3$^{+1.0}_{-1.3}$

### Comparisons using observables in top quark production

In the following, we investigate how the new pythia8 tunes describe the CMS $\mathrm{t}\overline{\mathrm{t}}$ data when different ME generators, namely powheg and mg5_amc, are employed. Both ME configurations use the NNPDF3.1 NNLO PDF with $\alpha _S (m_{\mathrm{Z}{}{}})=0.118$ and assume a top quark mass ($m_{\mathrm{t}{}{}}$) value of 172.5$\,\text {GeV}$.

In the powheg configuration, the ME heavy quark production mode [36, 39, 68] is used. In this configuration, powheg simulates inclusive $\mathrm{t}\overline{\mathrm{t}}$ production at NLO, where the first additional jet is computed at LO, while mg5_amc performs the calculation with up to 2 additional jets at NLO, with a third jet simulated at LO. The powheg generator scales the real emission cross section by a damping function that controls the ME-PS merging and that regulates the high-$p_{\mathrm {T}}$ radiation. The damping variable used in the powheg simulation is set to 1.379 times $m_{\mathrm{t}{}{}}$, a value derived from data at $\sqrt{s}=8\,\text {TeV}$ in the dilepton channel using a similar ME calculation and assuming the CP5 tune. The factorization and renormalization scales are assumed equal to the transverse mass of the top quark, $m_{\mathrm {T}} ^{\mathrm{t}{}{}} =\sqrt{\smash [b]{m_{\mathrm{t}{}{}} ^2+p_{\mathrm {T}} ^2}}$. The minimum $p_{\mathrm {T}}$ for the emission of light quarks in powheg is 0.8$\,\text {GeV}$. The pThard parameter is set to 0 and the powheg hardness criterion, defined by the pTdef option, is set to 1. The merging scale in mg5_amc  is set to 40$\,\text {GeV}$, and the threshold applied to regulate multijet MEs in the mg5_amc  FxFx merging procedure, is 20$\,\text {GeV}$.

**Fig. 19:**
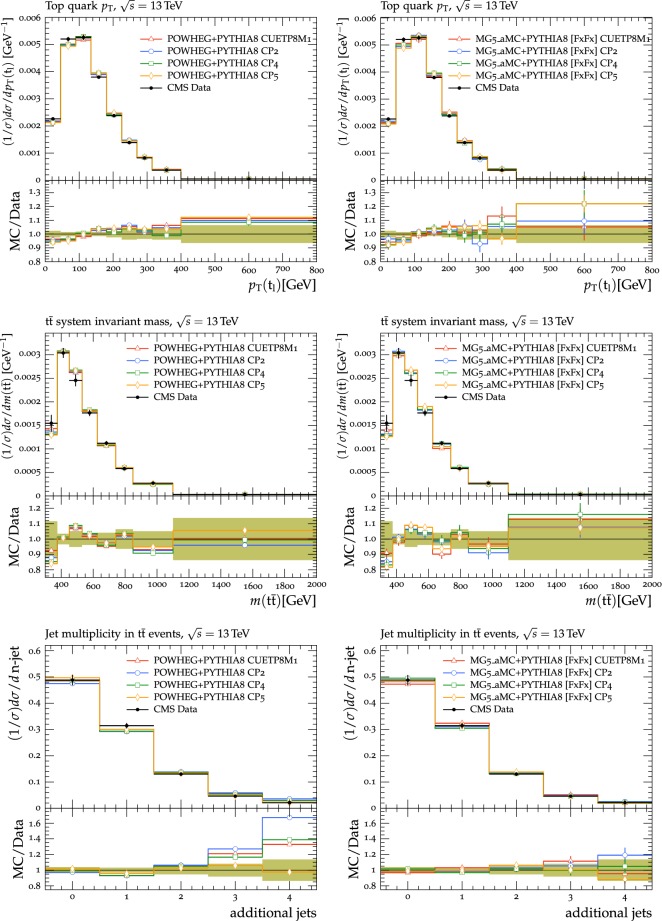
The normalized $\mathrm{t}\overline{\mathrm{t}}$ cross section in the lepton$+$jets channel, as a function of the transverse momentum of the top quark for leptonically decaying top quarks ($t_\ell$) (upper), the invariant mass of the $\mathrm{t}\overline{\mathrm{t}}$ system, *m*($\mathrm{t}\overline{\mathrm{t}}$) (middle), and in bins of number of additional jets (lower) from CMS $\sqrt{s}=13\,\text {TeV}$ analysis [69]. The data are compared with the predictions of powheg (left) and mg5_amc [FxFx] (right). In both cases, the PS simulation is done with the pythia8 tunes CUETP8M1, CP2, CP4, or CP5. Tunes CP1 and CP3 are not shown in the plot but present a similar behaviour as, respectively, tunes CP2 and CP4. The ratios of simulations to the data (MC/Data) are also shown, where the shaded band indicates the total experimental uncertainty in the data. Vertical lines drawn on the data points refer to the total uncertainty in the data. Vertical lines drawn on the MC points refer to the statistical uncertainty in the predictions. Horizontal bars indicate the associated bin width

Distributions [69] in the lepton$+$jets channel are compared to predictions from different tunes using various settings, namely, powheg  $+$ pythia8, and mg5_amc+pythia8 with FxFx merging [38], referred to as mg5_amc [FxFx] hereafter, with the CUETP8M1, CP2, CP4, and CP5 tunes. Figure 19 (upper panel) displays the normalized $\mathrm{t}\overline{\mathrm{t}}$ cross section in bins of $p_{\mathrm {T}}$ of the top quark decaying leptonically (${\mathrm{t}{}{}} _\ell$), in data and simulation. For all tunes, powheg  + pythia8 predictions have deviations below 10% with respect to the central values of the data. The central values of predictions from mg5_amc [FxFx] and data agree within $\approx$10% for $p_{\mathrm {T}} ({\mathrm{t}{}{}} _\ell )<400\,\text {GeV}$ and within $\approx$20% for higher $p_{\mathrm {T}}$.

Figure 19 (middle panel) shows the normalized $\mathrm{t}\overline{\mathrm{t}}$ cross section in bins of *m*($\mathrm{t}\overline{\mathrm{t}}$) in data and simulation. Predictions from powheg and mg5_amc [FxFx] with the new tunes describe the central values of the data reasonably well. Normalized $\mathrm{t}\overline{\mathrm{t}}$ cross sections in bins of number of additional jets in data and simulation in the lepton$+$jets channel at $\sqrt{s}=13\,\text {TeV}$ are shown in Fig. 19 (lower panel). The cross sections are compared with the predictions of powheg and of mg5_amc [FxFx]. The central values predicted by powheg  $+$ pythia8 are in good agreement with data when CP5 tune is used. The value of $\alpha _S ^\mathrm {ISR}(m_{\mathrm{Z}{}{}})$ in combination with the rapidity ordering for ISR in the pythia8 simulation affects the additional jet distribution in $\mathrm{t}\overline{\mathrm{t}}$ events. Predictions obtained from powheg  $+$ pythia8 overestimate the data when a high value of $\alpha _S ^\mathrm {ISR}(m_{\mathrm{Z}{}{}})\approx 0.13$ is used (CUETP8M1 and CP2 tunes) irrespective of rapidity ordering for ISR. It is observed that even when $\alpha _S ^\mathrm {ISR}(m_{\mathrm{Z}{}{}})=0.118$ is used, predictions from the CP4 tune overshoot the data at high jet multiplicities. A much better agreement of central values is obtained only when rapidity ordering for ISR is switched on in the pythia8 simulation and $\alpha _S ^\mathrm {ISR}(m_{\mathrm{Z}{}{}})=0.118$ is used as in the CP5 tune. Predictions from mg5_amc [FxFx]$+$ pythia8 with CUETP8M1, CP2, CP4, and CP5 tunes describe the central values of the data reasonably well.

**Fig. 20:**
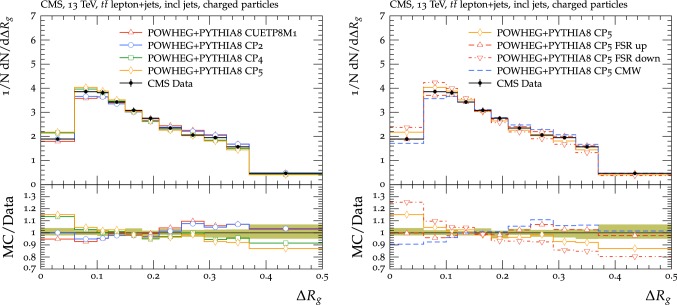
Comparison with the measurement [70] of the angle between two groomed subjets, $\varDelta R_g$ in $\mathrm{t}\overline{\mathrm{t}}$ events predicted by powheg  $+$ pythia8 for the different tunes. The data are compared to tunes CUETP8M1, CP2, CP4, and CP5 (left). Tunes CP1 and CP3 are not displayed but they present a similar behavior as tunes CP2 and CP4, respectively. The data are also compared to CP5, CP5 FSR up, CP5 FSR down, and CP5 with CMW rescaling (right). The ratios of simulations to the data (MC/Data) are also shown, where the shaded band indicates the total experimental uncertainty in data. Vertical lines drawn on the data points refer to the total uncertainty in the data. Horizontal bars indicate the associated bin width

Comparisons are also made using jet substructure observables in $\mathrm{t}\overline{\mathrm{t}}$ events in the lepton$+$jets channel using measurements by CMS at $\sqrt{s}=13\,\text {TeV}$ [70]. Figure 20 displays the comparisons using the distribution of the angle between two groomed subjets, $\varDelta R_g$, which is found to be the most sensitive to $\alpha _S ^\mathrm {FSR}(m_{\mathrm{Z}{}{}})$ [70]. The data are compared to simulations with the tunes CUETP8M1, CP2, CP4, and CP5, as well as CP5 FSR up ($\alpha _S ^\mathrm {FSR}(m_{\mathrm{Z}{}{}})=0.122$), CP5 FSR down ($\alpha _S ^\mathrm {FSR}(m_{\mathrm{Z}{}{}})=0.115$), and CP5 with CMW rescaling. It is observed that tunes with higher $\alpha _S ^\mathrm {FSR}(m_{\mathrm{Z}{}{}})$ (CUETP8M1, CP2, and CP5 FSR up) describe the data better. Tune CP5 with CMW rescaling resolves the discrepancy of CP5 at high $\varDelta R_g$, but worsens the description at $\varDelta R_g\sim 0.27$ compared to CP5. It should be noted that a fit to the $\varDelta R_g$ distribution using a b-enriched sample yields $\alpha _S ^\mathrm {FSR}(m_{\mathrm{Z}{}{}})=0.130^{+0.016}_{-0.020}$ [70] without CMW rescaling, while a fit to the distirubtion of the UE observable $\overline{p_{\mathrm {T}}}$ measured in $\mathrm{t}\overline{\mathrm{t}}$ events yields $\alpha _S ^\mathrm {FSR}(m_{\mathrm{Z}{}{}})=0.120\pm 0.006$ [22]. Therefore, in $\mathrm{t}\overline{\mathrm{t}}$ events, UE and jet substructure observables prefer different central $\alpha _S ^\mathrm {FSR}(m_{\mathrm{Z}{}{}})$ values, but they are compatible within uncertainties.

### Comparisons using observables in W and Z boson production

In this subsection, we present a validation of the new CMS UE tunes for observables measured in events with a W or Z boson in the final state at $\sqrt{s}=13\,\text {TeV}$. For the comparisons, we use predictions obtained with mg5_amc $+$ pythia8 at LO using the $k_{\mathrm {T}}$–MLM merging scheme, and at NLO using the FxFx merging scheme. The $k_{\mathrm {T}}$–MLM merging scale is set to 19$\,\text {GeV}$, while for FxFx the corresponding scale is set to 30$\,\text {GeV}$. In both cases the MEs include the final states with 0, 1, 2, and 3 partons, and up to 2 partons are calculated at NLO precision in the FxFx case. To ease the comparison of the different tunes, the same PDF, NNPDF3.1 NNLO, and $\alpha _S (m_{\mathrm{Z}{}{}}) = 0.118$ are used for the ME calculation independently of the tune.

**Fig. 21:**
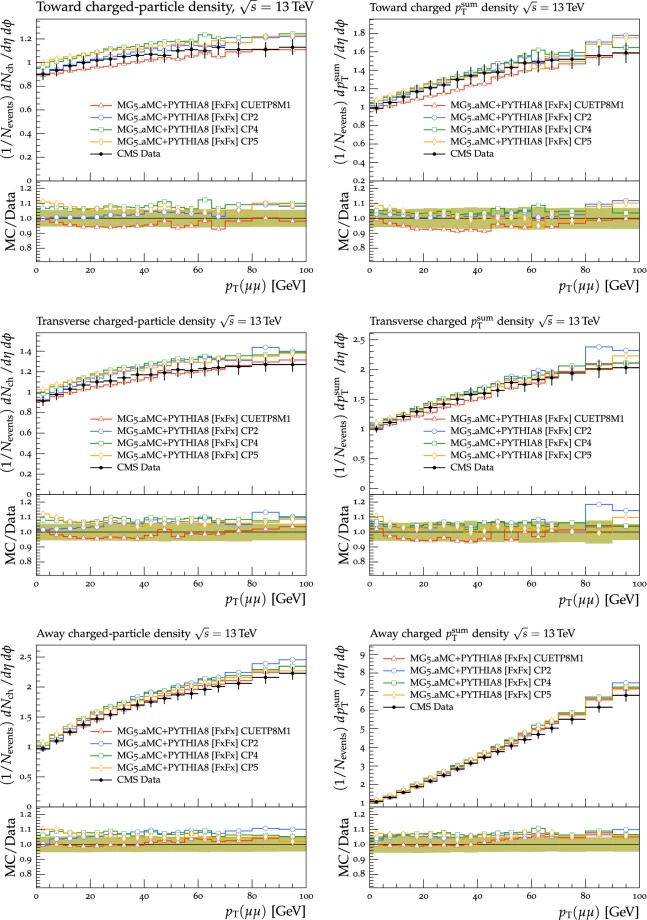
The charged-particle multiplicity (left) and $p_{\mathrm {T}} ^\text {sum}$ (right) in the toward (upper), transverse (middle), and away (lower) regions measured as a function of the Z boson $p_{\mathrm {T}}$ in Drell–Yan events at $\sqrt{s}=13\,\text {TeV}$ [21], and compared with the predictions obtained by an inclusive NLO ME calculated by mg5_amc, interfaced to the UE simulation of pythia8 with the CUETP8M1, CP2, CP4, and CP5 tunes. Tunes CP1 and CP3 are not shown in the plot but present a similar behaviour as, respectively, tunes CP2 and CP4. The ratios of simulations to the data (MC/Data) are also shown, where the shaded band indicates the total experimental uncertainty of the data. Vertical lines drawn on the data points refer to the total uncertainty in the data. Vertical lines drawn on the MC points refer to the statistical uncertainty in the predictions. Horizontal bars indicate the associated bin width

First, UE observables [21] in Drell–Yan events in an invariant mass window of 81–101$\,\text {GeV}$ around the Z boson peak for muonic decays are studied. The charged-particle density and transverse momentum sum are measured as a function of the Z boson $p_{\mathrm {T}}$ in the three regions introduced in Sect. 6.2: toward, away, and transverse. The regions are defined with respect to the Z boson direction. The measurements are compared with FxFx predictions obtained with the CUETP8M1, CP2, CP4, and CP5 tunes in Fig. 21. The measurements are, in general, well-described by all tunes.

The description of the cross section as a function of the jet multiplicity is also investigated in Z $+$jets [71] and W $+$jets [72] final states. The Z $+$jets measurement is restricted to the phase space where the two leptons have $p_{\mathrm {T}} >20\,\text {GeV}$ and $|y |<2.4$ and the dilepton mass lies in a $\pm 20\,\text {GeV}$ window around 91$\,\text {GeV}$. The momenta of the photons inside a cone of $\varDelta \text {R}<0.1$ are added to the lepton momentum in order to partly recover the energy lost by FSR. Jets are clustered using the anti-$k_{\mathrm {T}}$ algorithm with $R = 0.4$ and must satisfy the criteria $p_{\mathrm {T}} > 30\,\text {GeV}$ and $|y | < 2.4$. The distance between the selected leptons and the leading jet $\varDelta R(\ell ,\mathrm {j})$ must be greater than 0.4. For the W $+$jets measurement, the phase space is restricted by a transverse mass requirement, $m_{\mathrm {T}} > 50\,\text {GeV}$, and by requirements on the muon, $p_{\mathrm {T}} > 25\,\text {GeV}$ and $|y |<2.4$. In the Z $+$jets measurements the same clustering algorithm, the FSR recovery prescription described above, and the lepton jet separation requirement are applied.

**Fig. 22:**
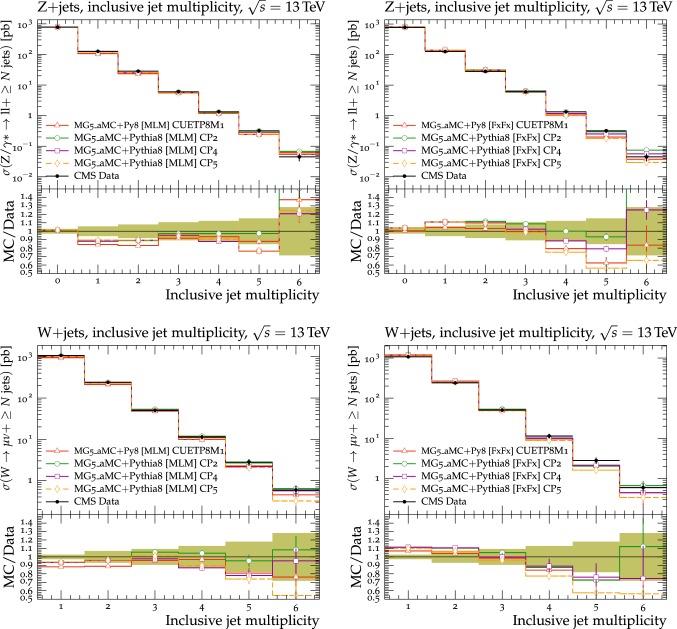
Comparison with the measurement [71, 72] of the inclusive jet multiplicity in Z $+$jets (upper) and W $+$jets (lower) events predicted by mg5_amc $+$ pythia8 with $k_{\mathrm {T}}$–MLM merging (left) and FxFx merging (right) for the different tunes. Tunes CP1 and CP3 are not shown in the plot but present a similar behaviour as, respectively, tunes CP2 and CP4. The ratios of simulations to the data (MC/Data) are also shown, where the shaded band indicates the total experimental uncertainty in the data. Vertical lines drawn on the data points refer to the total uncertainty in the data. Vertical lines drawn on the MC points refer to the statistical uncertainty in the predictions. Horizontal bars indicate the associated bin width

The comparisons of the jet multiplicities to various predictions are shown in Fig. 22. The measurement of the cross section inclusive in the number of jets, *N*, is not available for the W $+$jets analysis and the lower plots start at $N=1$. The $k_{\mathrm {T}}$–MLM predictions of the jet multiplicity have little sensitivity to the UE and PS tunes, so all the tunes provide a good description of this observable, with a slightly better agreement observed for the CP2 tune. In the case of the FxFx sample, the CP5 tune predicts fewer events with a jet multiplicity of more than four with respect to the measurement. The deficit increases for increasing jet multiplicities. The CUETP8M1 tune shows a similar behaviour, though.

**Fig. 23:**
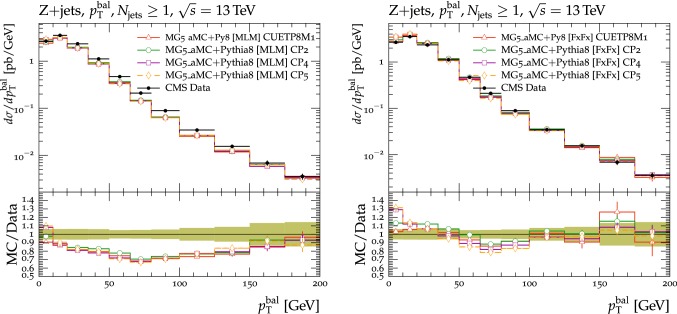
Comparison with the measurement [71] of the $p_{\mathrm {T}}$ balance predicted by mg5_amc $+$ pythia8 with $k_{\mathrm {T}}$–MLM merging (left) and FxFx merging (right) for the different tunes for events with at least one jet. Tunes CP1 and CP3 are not shown in the plot but they present a similar behaviour as tunes CP2 and CP4, respectively. The ratios of simulations to the data (MC/Data) are also shown, where the shaded band indicates the total experimental uncertainty in the data. Vertical lines drawn on the data points refer to the total uncertainty in the data. Vertical lines drawn on the MC points refer to the statistical uncertainty in the predictions. Horizontal bars indicate the associated bin width

Predictions using the new CMS UE tunes are also compared with the $p_{\mathrm {T}}$ balance between the Z boson and the jets with $p_{\mathrm {T}} > 30\,\text {GeV}$ and $|y |<2.4$ using the variable $p_{\mathrm {T}}$ $^\text {bal} = |{\vec p}_{\mathrm {T}} ({\mathrm{Z}{}{}}) + \sum _{\text {jets}} {\vec p}_{\mathrm {T}} (\mathrm {j}_i) |$ [71]. This variable is sensitive to PS and UE. The comparison is shown in Fig. 23 for events with at least one jet. Differences between the tunes are significant only in the region below $\approx$20$\,\text {GeV}$. The discrepancy in this region for the FxFx samples indicates that the distribution peaks at lower values for CP4 and CP5 than in data.

**Fig. 24:**
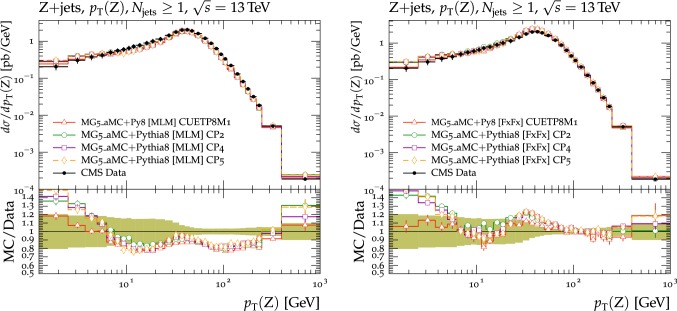
Comparison with the measurement [71] of the $p_{\mathrm {T}}$ (Z) predicted by mg5_amc $+$ pythia8 with $k_{\mathrm {T}}$–MLM merging (left) and FxFx merging (right) for the different tunes. Tunes CP1 and CP3 are not shown in the plot but they present a similar behaviour as tunes CP2 and CP4, respectively. The ratios of simulations to the data (MC/Data) are also shown, where the shaded band indicates the total experimental uncertainty in data. Vertical lines drawn on the data points refer to the total uncertainty in the data. Vertical lines drawn on the MC points refer to the statistical uncertainty in the predictions. Horizontal bars indicate the associated bin width

Results of Ref. [71] are also used to validate the description of the transverse momentum of the weak vector boson in ${\mathrm{Z}{}{}}$ $+$ $\ge 1$ jet events. The comparison is shown in Fig. 24. The new tunes provide similar descriptions for this distribution. Predictions using $k_{\mathrm {T}}$–MLM achieve a poor agreement with the data, independently of the UE tune, with respect to FxFx, which is able to describe the transverse momentum of the Z boson at $p_{\mathrm {T}} >10\,\text {GeV}$. The region below 10$\,\text {GeV}$ is poorly described for both FxFx and $k_{\mathrm {T}}$–MLM and the new tunes, but is well-described by predictions using the CUETP8M1 tune.

To summarize the study of weak vector boson production, the CP2, CP4, and CP5 tunes provide similar descriptions of the UE observables with a reasonable agreement with the data. In general, the CP2 tune performs better in describing variables such as $p_{\mathrm {T}}$ $^{\text {bal}}$ and $p_{\mathrm {T}}$ (Z). For the jet multiplicity, the CP2 and CP4 tunes are equally good in describing the measurement, whereas CP5 tends to undershoot the PS dominated region with at least five jets with a significance of 3.5 standard deviations.

## Summary and conclusions

A new set of tunes for the underlying-event (UE) simulation in the pythia8 event generator is obtained by fitting various measurements sensitive to soft and semihard multipartonic interactions at different collision energies. To derive these tunes, the leading order (LO), next-to-leading order (NLO), or next-to-next-to-leading order (NNLO) versions of the NNPDF3.1 parton distribution function (PDF) set for the simulation of the underlying-event components are used. In these tunes, the values of the strong coupling, $\alpha _S (m_{\mathrm{Z}{}{}})$, used for the simulation of hard scattering, initial- and final-state radiation, and multiple-parton interactions are chosen consistent with the order of the PDF used. In the LO NNPDF3.1 set, $\alpha _S (m_{\mathrm{Z}{}{}}) = 0.130$, whereas for the NLO and NNLO NNPDF3.1 sets, $\alpha _S (m_{\mathrm{Z}{}{}}) = 0.118$. In general, the combination of contributions from multiple-parton interactions and parton-shower emissions is crucial to give a good description of variables measured in soft-collision events. The infrared threshold is relatively independent of center-of-mass energy when using NLO or NNLO PDF sets. Irrespective of the specific PDF used, predictions from the new tunes reproduce well the UE measurements at center-of-mass energies $\sqrt{s}=1.96$ and 7$\,\text {TeV}$. A significant improvement in the description of UE measurements at 13$\,\text {TeV}$ is observed with respect to predictions from old tunes that were extracted using data at lower collision energies. For the first time, predictions based on higher-order PDF sets are shown to give a reliable description of minimum-bias (MB) and UE measurements, with a similar level of agreement as predictions from tunes using LO PDF sets.

Predictions of the new tunes agree well with the data for MB observables measured at pseudorapidities in the central ($|\eta |< 2.4$) and forward ($3.2<|\eta |< 4.7$) regions. The new CMS tunes simultaneously describe the number of charged particles produced in diffractive processes and MB collisions. Neither the new CMS tunes nor the CUETP8M1 tune describe the very forward region ($-6.6<\eta <-5.2$) well.

Measurements sensitive to double-parton scattering contributions are reproduced better by predictions using the LO PDF set in the UE simulation, without rapidity ordering of the initial-state shower.

The UE simulation provided by the new tunes can be interfaced to higher-order and multileg matrix element generators, such as powheg and mg5_amc, without degrading the good description of UE observables. Such predictions also reproduce well observables measured in multijet final states, Drell–Yan, and top quark production processes. The central values of the normalized $\mathrm{t}\overline{\mathrm{t}}$ cross section in bins of the number of additional jets predicted by powheg  $+$ pythia8 overestimate the data when a high value of $\alpha _S ^\mathrm {ISR}(m_{\mathrm{Z}{}{}})\gtrsim 0.130$ is used (CMS pythia8 CP1 and CP2 tunes). Even when $\alpha _S ^\mathrm {ISR}(m_{\mathrm{Z}{}{}})=0.118$ is used, the CP4 tune overestimates the data at high jet multiplicities. This is cured by the rapidity ordering of the initial-state shower (CP5 tune). Measurements of azimuthal dijet correlations are also better described when a value of $\alpha _S ^\mathrm {ISR}(m_{\mathrm{Z}{}{}})=0.118$ is used in predictions obtained with powheg merged with pythia8.

Comparisons with LEP event-shape observables and the distribution of the angle between two groomed subjets ($\varDelta R_g$) in $\mathrm{t}\overline{\mathrm{t}}$ events at the LHC show that in ME-PS merged configurations CMW rescaling is disfavored. It is also found that $\varDelta R_g$ is better described by tunes with $\alpha _S ^\mathrm {FSR}(m_{\mathrm{Z}{}{}})$ higher than $\sim$0.120 while LEP event-shape observables and UE event observables in $\mathrm{t}\overline{\mathrm{t}}$ events prefer a central value $\sim$0.120 [22].

All of the new CMS tunes are supplied with their eigentunes, which can also be used to determine the uncertainties associated with the theoretical predictions. We show that predictions using the new tunes based on PDFs determined at LO, NLO, and NNLO agree reasonably well with the measurements, and that the new tunes can also be applied to LO and NLO calculations merged with parton showers, multiple-parton interactions, and hadronization.

## Figures and Tables

**Fig. 25:**
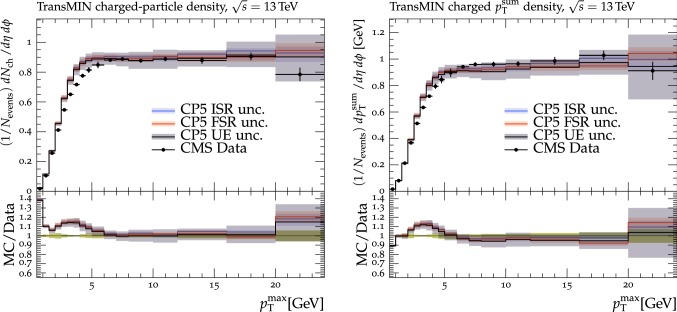
The variations allowed by the CP5 tune when $\alpha _S ^\mathrm {ISR}(m_{\mathrm{Z}{}{}})$ (blue band) and $\alpha _S ^\mathrm {FSR}(m_{\mathrm{Z}{}{}})$ (red band) are left free in the fit for charged-particle (left) and charged $p_{\mathrm {T}} ^\text {sum}$ (right) density in the transMIN region at $\sqrt{s}=13\,\text {TeV}$. Vertical lines drawn on the data points refer to the total uncertainty in the data. The grey band represents the total UE uncertainty for the tune CP5. Horizontal bars indicate the associated bin width

## Data Availability

This manuscript has no associated data or the data will not be deposited. [Author’s comment: Release and preservation of data used by the CMS Collaboration as the basis for publications is guided by the CMS policy as written in its document “CMS data preservation, re-use and open access policy” (https://cmsdocdb.cern.ch/cgi-bin/PublicDocDB/RetrieveFile?docid=6032&filename=CMSDataPolicyV1.2.pdf&version=2).]

## A Tables of tune uncertainties

This section provides the values of the parameters corresponding to the uncertainties when the new CMS pythia8 tunes are used. The tune uncertainty is obtained by extracting the eigentunes, which are defined by a change in the $\chi ^2$ of the fit that equals the absolute $\chi ^2$ value obtained in the tune. The eigentunes refer to the variations of the tunes along each of the maximally independent directions in the parameter space, obtained by using the covariance matrix in the region of the best tune. The number of directions defined in the parameter space equals the number of free parameters *n* used in the fit and results into 2*n* parameter variations, i.e., eigentunes. These variations represent a good set of systematic uncertainties in the given tune.

The estimations of the tune uncertainties, which have 2*n* parameter variations, i.e., 10 for the new CMS pythia8 tunes, are very time consuming in analyses, since for each variation separate samples must be produced. Therefore, a lower number of variations is preferred. Hence, two variations, one “up” and one “down”, are defined. For the definition of the two variations, predictions using the parameters of the eigentunes are implemented for the UE observables at $\sqrt{s}=13\,\text {TeV}$ and their differences with respect to the central predictions are added in quadrature. This procedure is applied in each bin and tune uncertainties are estimated without any correlation across the different bins. Positive differences between central predictions and tune variations define the upper edge of the bin-by-bin uncertainty, while negative differences define the lower edge of the bin-by-bin uncertainty. By following the same approach used for the extraction of the central values of the new CMS pythia8 tunes, the upper edge is fitted to obtain the “up variation”, while the “down variation” is obtained by fitting the lower edge. The parameters of the up- and down-variations are listed in Table 6 for the tunes using LO PDF sets, and in Table 7 for the tunes using (N)NLO PDF sets. We checked that for a wide range of MB and UE observables at $\sqrt{s}=13\,\text {TeV}$ predictions from up- and down-variations, obtained by including the full set of eigenvalues, reproduce well the upper and the lower edge of the predictions. Hence, tune uncertainties estimated by evaluating predictions of up- and down-variations represent a reliable way of estimating the systematic uncertainties in the tunes. The correlation matrix for the fit of the CP5 tune is displayed in Table 8. It is retrieved by evaluating the correlation of the parameter variations obtained in the eigentunes.

**Table 6:** Parameters of the “up-” and “down-”variation eigentunes for the pythia8 CP1, and CP2 tunes

pythia8 parameter	CP1	CP1	CP2	CP2
	Up	Down	Up	Down
MultipartonInteractions:pT0Ref ($\text {GeV}$ )	2.30	2.40	2.34	2.33
MultipartonInteractions:ecmPow	0.15	0.15	0.14	0.14
MultipartonInteractions:coreFraction	0.51	0.39	0.51	0.23
MultipartonInteractions:coreRadius	0.58	0.60	0.41	0.34
ColourReconnection:range	8.31	8.50	1.46	2.56

**Table 7:** Parameters of the “up-” and “down-”variation eigentunes for the pythia8 CP3, CP4, and CP5 tunes

pythia8 parameter	CP3	CP3	CP4	CP4	CP5	CP5
	Up	Down	Up	Down	Up	Down
MultipartonInteractions:pT0Ref ($\text {GeV}$ )	1.48	1.54	1.48	1.54	1.41	1.46
MultipartonInteractions:ecmPow	0.02	0.02	0.02	0.02	0.03	0.03
MultipartonInteractions:coreFraction	0.35	0.25	0.36	0.33	0.43	0.73
MultipartonInteractions:coreRadius	0.49	0.35	0.58	0.57	0.67	0.69
ColourReconnection:range	8.15	3.96	7.93	6.88	4.88	4.69

**Table 8:** The correlation matrix, retrieved when extracting the CP5 tune. This is obtained by evaluating the correlation values of the parameter variations obtained in the eigentunes

	pT0Ref	ecmPow	coreFraction	coreRadius	range
pT0Ref	1.00	$-$0.21	$-$0.19	$-$0.19	0.15
ecmPow	$-$0.21	1.00	0.30	0.69	$-$0.21
coreFraction	$-$0.19	0.30	1.00	0.32	$-$0.64
coreRadius	$-$0.19	0.69	0.32	1.00	$-$0.52
range	0.15	$-$0.21	$-$0.64	$-$0.52	1.00

Variations of the values of the ISR and FSR are also studied, in order to check the consistency of the selected $\alpha _S ^\mathrm {ISR}(m_{\mathrm{Z}{}{}})$ and $\alpha _S ^\mathrm {FSR}(m_{\mathrm{Z}{}{}})$ values selected for the tunes and to estimate the allowed range of $\alpha _S ^\mathrm {ISR}(m_{\mathrm{Z}{}{}})$ and $\alpha _S ^\mathrm {FSR}(m_{\mathrm{Z}{}{}})$ values in the PS using the CP5 tune. Starting from tune CP5, the value of $\alpha _S ^\mathrm {ISR}(m_{\mathrm{Z}{}{}})$ is fitted to UE observables measured by CMS at $\sqrt{s}=13\,\text {TeV}$. The same procedure is repeated when $\alpha _S ^\mathrm {FSR}(m_{\mathrm{Z}{}{}})$ is fitted. The parameters obtained from the fits are shown in Table 9, along with the up and down variation.

**Table 9:** “Up” and “Down” ISR and FSR variations for CP5 when $\alpha _S ^\mathrm {ISR}(m_{\mathrm{Z}{}{}})$ or $\alpha _S ^\mathrm {FSR}(m_{\mathrm{Z}{}{}})$ is treated as a free parameter

pythia8 parameter	Central	Up	Down	$\chi ^2$/dof
$\alpha _S ^\mathrm {ISR}(m_{\mathrm{Z}{}{}})$ value	0.121	0.128	0.114	0.75
$\alpha _S ^\mathrm {FSR}(m_{\mathrm{Z}{}{}})$ value	0.119	0.122	0.115	0.78

Figure 25 shows the predictions of the CP5 tune, with the corresponding variation bands relative to the UE parameters, and the $\alpha _S ^\mathrm {ISR}(m_{\mathrm{Z}{}{}})$ and $\alpha _S ^\mathrm {FSR}(m_{\mathrm{Z}{}{}})$ values for the charged-particle and $p_{\mathrm {T}} ^\text {sum}$ densities at $\sqrt{s}=13\,\text {TeV}$ in the transMIN region.
